# Green and sustainable approaches for the Friedel–Crafts reaction between aldehydes and indoles

**DOI:** 10.3762/bjoc.20.36

**Published:** 2024-02-22

**Authors:** Periklis X Kolagkis, Eirini M Galathri, Christoforos G Kokotos

**Affiliations:** 1 Laboratory of Organic Chemistry, Department of Organic Chemistry, National and Kapodistrian University of Athens, Athens, 15771, Greece

**Keywords:** aldehyde, BIMs, Friedel–Crafts reaction, green chemistry, indole

## Abstract

The synthesis of indoles and their derivatives, more specifically bis(indolyl)methanes (BIMs), has been an area of great interest in organic chemistry, since these compounds exhibit a range of interesting biological and pharmacological properties. BIMs are naturally found in cruciferous vegetables and have been shown to be effective antifungal, antibacterial, anti-inflammatory, and even anticancer agents. Traditionally, the synthesis of BIMs has been achieved upon the acidic condensation of an aldehyde with indole, utilizing a variety of protic or Lewis acids. However, due to the increased environmental awareness of our society, the focus has shifted towards the development of greener synthetic technologies, like photocatalysis, organocatalysis, the use of nanocatalysts, microwave irradiation, ball milling, continuous flow, and many more. Thus, in this review, we summarize the medicinal properties of BIMs and the developed BIM synthetic protocols, utilizing the reaction between aldehydes with indoles, while focusing on the more environmentally friendly methods developed over the years.

## Review

### Medicinal properties

**Scheme 1:**
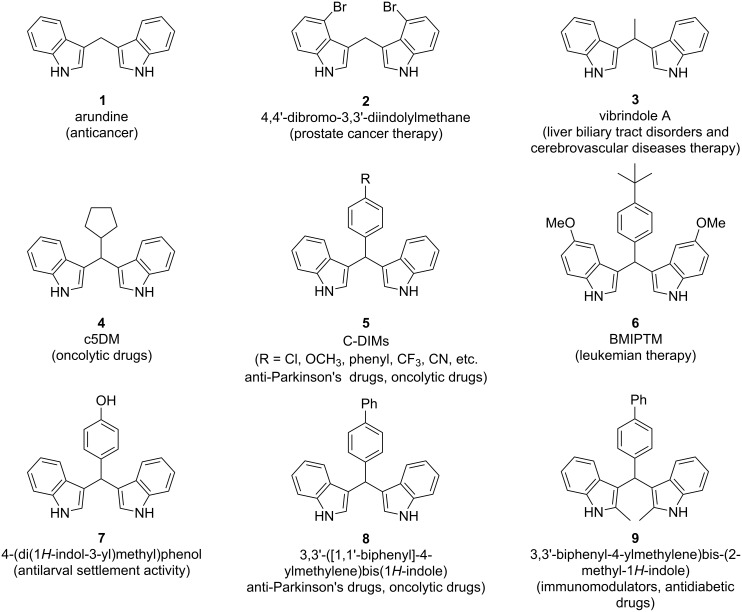
Examples of BIMs used for their medicinal properties.

In recent years, diindolylmethane (DIM, **1**) and its derivatives known as bis(indolyl)methanes (BIMs) have found increased use either as standalone medicine or in combination with other compounds for their bactericidal and fungicidal properties (Scheme 1). BIMs are natural products that are found in certain marine species of sponges and have also been isolated from cruciferous vegetables, as hydrolysis products from the metabolism of glucosinolates [1].

In 2017, Müller and her research team showcased the ability of BIMs to operate as anti-inflammatory drugs by acting as GPR84 agonists [2]. GPR84 is a protein-coupled receptor found in immune cells, which regulates a number of inflammatory processes, which can lead to inflammatory bowel disease or Alzheimer’s disease. Therefore, GPR84 agonists are used as medicine for the treatment of various inflammatory diseases, but they have been also linked to the reduction of leukemia cells, since the continuous expression of the GPR receptor can aggravate the symptoms of myeloid leukemia. In contrast to most GPR84 agonists which contain long alkyl chains, BIMs are not lipophilic molecules, which allow them to bind to the GPR receptor via an allosteric binding site and modulate GPR84’s rate of expression [2].

One application of BIMs, as referenced above, that is gaining attraction is the treatment of Crohn’s disease, which is an inflammatory bowel disease (IBD). Kang and his research team monitored the production of IL-8 and IL-1β, which are cytokines produced in response to inflammation, and they observed the reduction in the concentration of these cytokines, as well as, increased intestinal permeability and improved expression of tight junction proteins that control the polarity of cells when using DIM in Caco-2 human cells [3].

BIMs have also been linked with the therapy of various cancers with one of their most common applications being the treatment of breast cancer. Studies have shown that breast cancer is tied to the ratio of 16α-hydroxyestrone (16αOHE1) and 2-hydroxyestrone (2OHE1), which are products of the metabolism of the estrogen 17β-estradiol. BIMs can shift the process of estrogen metabolism towards the production of 2OHE1, thus reducing the risk for estrogen-sensitive cancers, especially in combination with tamoxifen, which is an estrogen receptor modulator used in the treatment of breast cancer [4]. BIMs increase the efficacy of the antitumor activity of tamoxifen and reduce the side-effects that it can cause in the more sensitive subgroups of patients. Specifically, when DIM was used in tandem with tamoxifen, the ratio between 2OHE1/16αOHE1 increased up to 229%, as well as the concentration of the sex hormone binding globulin (SHBG) that inhibits the growth of breast cancer cells [4]. DIM has also been found to initiate the expression of tumor suppressing proteins (ATM, p21, p27kip), which control cell growth and protect cells against ionizing radiation, which can cause DNA mutations, decreasing the overall risk of breast cancer [1,5–6].

The cytotoxicity of DIM and BIMs in general extends to other types of cancers as well, such as prostate cancer by being an androgen receptor (AR) agonist in LNCaP prostate cancer cells [6]. DIM controls cell growth rates in AR-negative cells, while also targeting the mitochondria inducing apoptosis, which alleviates some of the symptoms of prostate cancer. The same apoptotic function can be observed in colon and pancreatic cancer, since BIMs (specifically DIM-C-pPhOCH_3_ (**5**)) can act as a Nur77 (Nuclear Receptor 4A1) antagonist, which modulates the life cycle of cells [6]. The correlation between lower lung cancer risk and the consumption of cruciferous vegetables has also been showcased, due to the function of BIMs as oxidative stress inhibitors; however, the specific mechanism of action has yet to be determined [1,6].

This capability of BIMs to act as Nur77 antagonists, has resulted in their examination as potential anti-Parkinson’s disease drugs by halting the growth of brain tumor cells and inhibiting the expression of inflammatory genes [7]. Since they can be administered orally, and they display satisfying distribution to the brain and plasma without leading to serious unwanted side effects, they have entered advanced stages of clinical trials as neuroprotective agents, presenting an attractive alternative to traditional anti-inflammatory and anti-Parkinson’s disease drugs [7].

With the increased drug resistance of bacteria to modern medicine, BIMs have emerged as an interesting alternative, due to their antibacterial and antiviral properties. BIMs function as selective antibacterial agents against several virulent *Escherichia coli* (*E. coli*) strains, which can cause many gut and urinary tract infections. They act by damaging DNA molecules and inhibiting their replication in bacteria, while also targeting the proteins that are responsible for bacterial cell division, such as FtsZ, which reduces the rate of bacterial growth in a reversible manner. BIMs have also been implemented against methicillin-resistant *Staphylococcus aureus* (MRSA), which is a drug-resistant bacterium that is usually encountered in health-care related environments [8]. The mechanism of action involves the binding of BIMs to the penicillin-restricting protein PBP2a which inhibits the biosynthesis of the bacterial cell wall, making the treatment feasible without any toxicity to human cells [9–10].

The applications of BIMs have also been extended to agriculture, since there is a need for the development of new greener fungicidal and antiviral agents that can combat the more common plant diseases [11]. One instance of their antiviral property being implemented in drug development involves the treatment of tobacco mosaic virus (TMV), which infects a great number of agricultural plants, causing great harm to production by evolving to resist most of the existing drugs. Thus, BIMs have emerged as a new natural alternative class of antiviral agents, surpassing commonly used drugs such as ribavirin that has been observed to damage the DNA strands of TMV, inhibiting their ability to multiply and cause damage to the plant. At the same time, they also display fungicidal activities against more than fourteen types of common fungi, such as *Phytophthora alternaria*, based on their DNA damaging properties with the thiourea derivative, showing the highest fungicidal activity [11].

### Brønsted or Lewis acid catalysis – conventional synthetic methods

The indole moiety is part of many natural products, agrochemicals, and pharmaceuticals. In medicinal chemistry, indole and its derivatives are considered important compounds, since they exhibit valuable pharmaceutical and biological activities. Among indole derivatives, bis(indolyl)methanes (BIMs) are profoundly interesting, due to their wide range of pharmaceutical properties. The most common approach involves the electrophilic substitution of various aldehydes and ketones by indoles, utilizing either protic or Lewis acids as catalysts. In 2010, Shiri published a review, where the majority of the acidic catalysts that have been employed for the synthesis of these compounds were presented [12]. Since then, various alternative acids have been applied including protic acids, such as silica-bonded S-sulfonic acid [13], polyvinylsulfonic acid (PVSA) [14], kaolin-supported H_2_SO_4_ [15], polyvinylpolypyrrolidone-supported triflic acid (PVPP^.^OTf) [16], ascorbic acid [17], phosphoric acid [18], benzenesulfonic acid [19], chitosan–SO_3_H (CTSA) [20], phthalimide-*N*-sulfonic acid (PISA) [21] and SiO_2_-KHSO_4_ [22] as well as Lewis acids, such as FeCl_3_ [23–24], RuCl_3_·3H_2_O [25], AgNO_3_ [26], glycerol and [Fe(III)-(salen)]Cl [27], Fe(DS)_3_·*n*H_2_O [28], Sc(OTf)_3_ [29], B(C_6_F_5_)_3_ or PhSiCl_3_ [30] and Cp_2_TiCl_2_ [31]. However, most of these reactions face some serious drawbacks, such as the requirement of large quantities of the catalyst due to present moisture or formation of adducts with the substrate, long reaction times, lower yields, and production of large amounts of toxic waste during work-up.

**Scheme 2:**
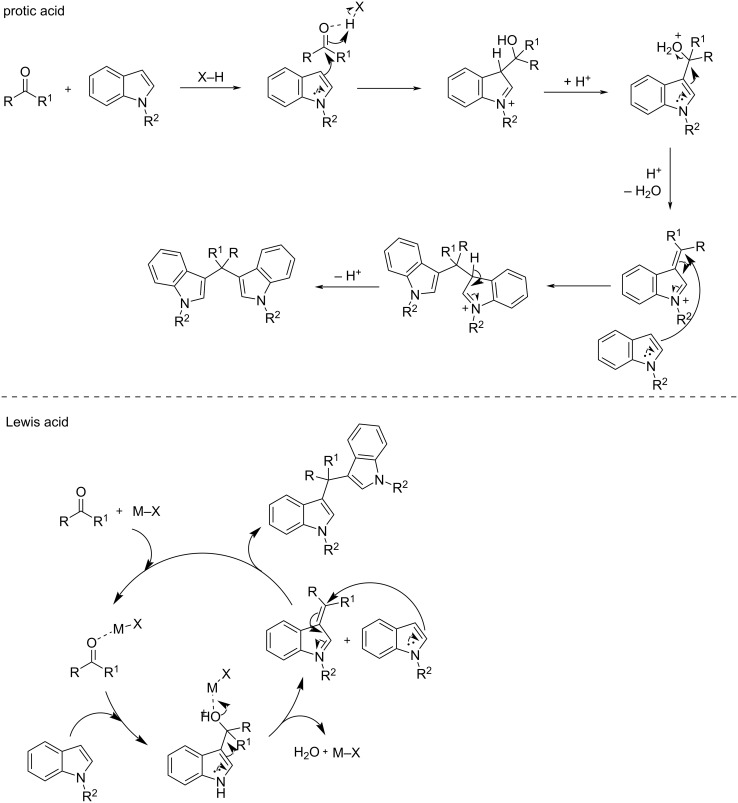
Mechanisms for the synthesis of BIMs using protic or Lewis acids as catalysts.

The general mechanisms of protic acid and Lewis acid-catalyzed syntheses of BIMs is shown in Scheme 2. In either case, the first step involves the activation of the carbonyl group by the catalyst. This renders it susceptible to a nucleophilic attack from the indole, leading to the formation of the intermediate product. Subsequently, a second nucleophilic attack occurs by another molecule of indole, yielding the final BIM product. The difference between the two mechanistic pathways is the nature of activation of the carbonyl group. Protic acids induce the protonation of the carbonyl group of the aldehyde or ketone, enhancing its electrophilic character. Whereas, Lewis acid catalysts bind to the heteroatom of the carbonyl group, lowering its LUMO energy, by withdrawing electron-density through a variety of covalent interactions.

### Green and sustainable approaches

#### Solvent-free processes

Alternative processes, that limit environmental pollution and toxic byproducts, came to the forefront of research for the introduction of novel synthetic pathways in organic chemistry [32–34]. Common organic syntheses require the use of harmful chemicals, such as toxic solvents, hazardous reagents, catalysts and reaction conditions, which contribute to environmental pollution and soil degradation [35–36]. Wanting to enhance the sustainability and viability of these synthetic protocols, organic chemists have been opting towards the use of greener catalysts and solvents in drug development.

Chemists dream to perform reactions under solvent-free conditions, which provide a greener approach towards organic transformations. Nowadays, the use of solvent-free reaction conditions has been introduced as a popular alternative to common organic solvents for many different organic transformations. The lack of an organic solvent can result in improved yields and reaction rates, more facile work-up processes and reduced waste, which are among the goals of green chemistry.

Organocatalysis is the acceleration of chemical reactions with the use of small organic compounds, which do not contain any amounts of enzyme or inorganic elements [37–39]. The benefits of solid acid catalysis render them as an appealing choice, compared to their liquid counterparts, due to their recyclability, ease of handling, and low cost [40]. Carbon-based solid acid catalysts especially are an interesting catalyst class, because they display low corrosiveness, toxicity, and higher catalytic activity, while also being insoluble in most organic solvents. The large amount of strong acidic sites that are on the carbon-based solid acids enhance their catalytic ability, compared to traditional Lewis and protic acids [41–43].

The most common examples in literature for the reaction of the synthesis of BIMs under solvent-free conditions utilize either protic acids, such as camphorsulfonic acid (CSA) [44], diammonium hydrogen phosphate (DAHP) [45], Amberlyst 15 [46], P_2_O_5_/MeSO_3_H [47], *p*-sulfonic acid calix[4]arene [48], xanthan sulfuric acid (XSA) [49], H_5_PW_10_V_2_O_40_/pyridino-santa barbara amorphous-15 (SBA-15) [50], TiO_2_-SO_4_^2−^ [51–52], humic acid [53] or Lewis acids, such as *N*-bromosuccinimide (NBS) [54], silica chloride [55], Ph_3_CCl [56], ZnO [57], La(NO_3_)_3_·6H_2_O [58], V(HSO_4_)_3_ [59], Cu(ClO_4_)_2_·6H_2_O [60], Fe/Al pillared clay [61], trimethylsilyl chloride (TMSCl) [62], BiCl_3_-loaded montmorillonite K10 [63–67], ZrO_2_-MgO [68] or CaO [69]. Silica gel is an intriguing solid support, since it is a low cost, commercially available and non-hazardous support, that can be employed in tandem with various traditional catalysts [70]. Some examples in literature are ZrOCl_2_·8H_2_O/SiO_2_ [71], P_2_O_5_/SiO_2_ [72], LiHSO_4_/SiO_2_ [73], (PhCH_2_PPh_3_)^+^Br^−^/SiO_2_ [74], H_2_SO_4_/SiO_2_ [75], ZnCl_2_/SiO_2_ [76], heteropoly-11-tungsto-1-vanadophosphoric acid, H4[PVV W11O40] (HPV) (20%) supported on natural clay (HPVAC-20) [77], V_2_O_5_/SiO_2_ [78], strongly acidic cation exchange resin (Seralite SRC-120) [79] or HCl/SiO_2_ [80].

**Scheme 3:**
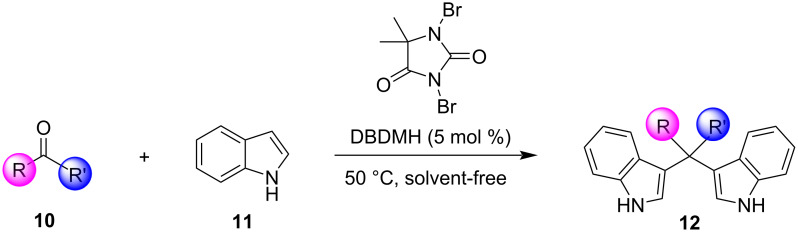
Synthesis of bis(indolyl)methanes using DBDMH.

**Scheme 4:**
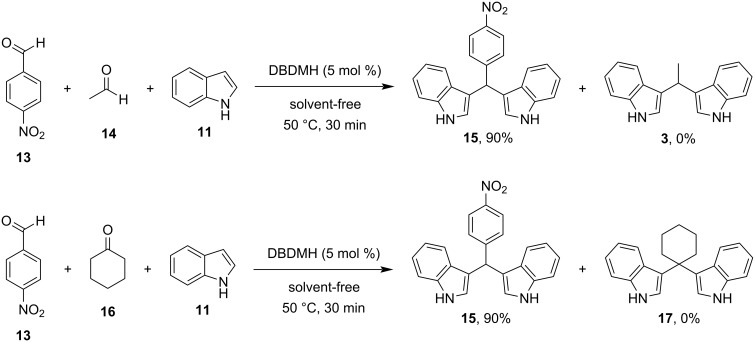
Competition experiments and synthesis of bis(indolyl)methanes using DBDMH.

Among all the protocols mentioned above, it is worth mentioning that Hojati et al., in 2013, developed a simple, novel and efficient procedure for the synthesis of BIMs, utilizing 1,3-dibromo-5,5-dimethylhydantoin (DBDMH) as the catalyst (Scheme 3). DBDMH is an *N*-halo-reagent, which has found widespread applications in industrial processes, due to its economic advantages. DBDMH is a well-known brominating and oxidizing agent that has recently gained special attention as a highly efficient, commercially available and inexpensive homogeneous catalyst [81]. After optimization, it was found that when employing a molar ratio of benzaldehyde, indole and DBDMH of 1:2:0.05, under solvent-free conditions at 50 °C for 50 min, the product was obtained in 90% yield. To emphasize the role of DBDMH, when the reaction was performed without DBDMH, no product was observed. The generality of this protocol was also tested by employing various aromatic aldehydes, which formed the corresponding BIMs in good to excellent yields (70–95%). It is important to mention that electron-withdrawing groups led to enhanced reaction rates and product yields, compared to their electron-donating substituents. However, aliphatic aldehydes and ketones displayed significantly lower reactivities in this methodology, affording low product yields, which limits some applications. The selectivity of this protocol was also investigated (Scheme 4). It was surprisingly observed that aromatic aldehydes produced the corresponding BIM as the major product in the presence of other substrates, rendering this protocol applicable for the chemoselective conversion of aromatic aldehydes to corresponding bis(indolyl)methanes in the presence of aliphatic aldehydes and ketones [81].

**Scheme 5:**
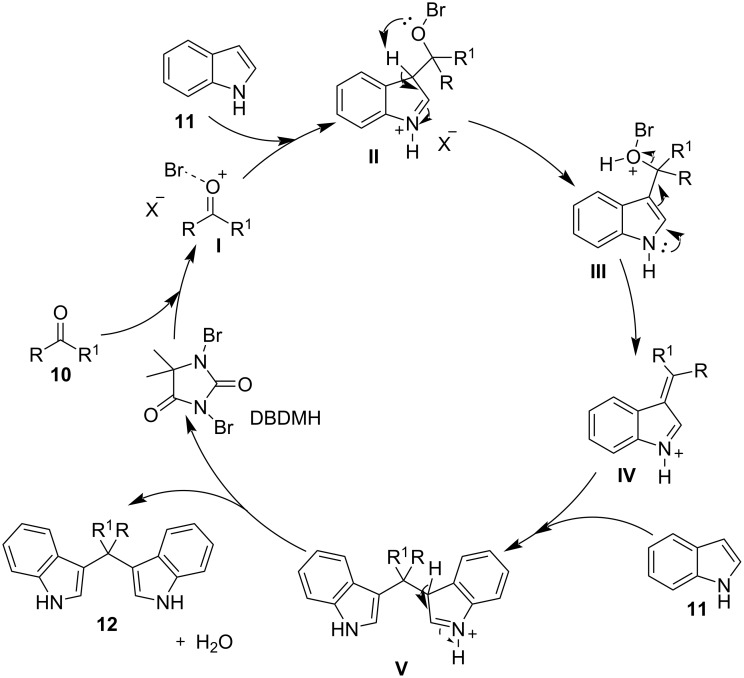
Proposed mechanism for formation of BIM of using DBDMH.

The proposed reaction mechanism for this protocol is showcased in Scheme 5. At the beginning of the reaction, the bromide ion activates the carbonyl group of the aldehyde, enabling a nucleophilic attack by a molecule of indole, producing the azafulvenium salt **IV**. The azafulvenium salt is formed, only when utilizing aromatic aldehydes, as opposed to aliphatic aldehydes, which cannot produce a stable conjugated system. Finally, another nucleophilic attack by a second molecule of indole to **IV** is occurring, forming the desired BIM **12**, while simultaneously releasing the catalyst, rendering it available for another catalytic cycle [80].

#### Halogen bonding processes

Recently, halogen bonding (XB) interactions have emerged as an interesting alternative to hydrogen bonding, which constitute an indispensable type of non-covalent interaction utilized in several catalytic approaches [81–88]. In 2008, Bolm introduced the use of perfluoroiodoalkanes as XB catalysts and the field gained widespread attention as an intriguing tool in the catalysis of various organic transformations that were previously considered unfeasible [89].

**Scheme 6:**
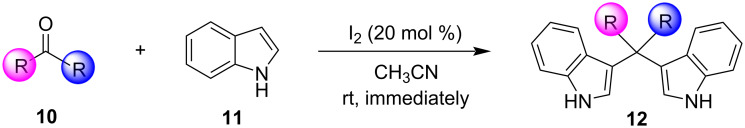
Synthesis of bis(indolyl)methanes using I_2_.

**Scheme 7:**
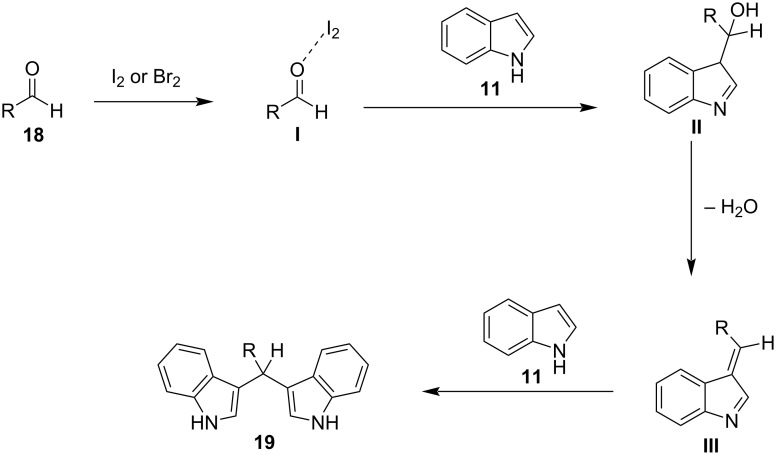
General reaction mechanism upon halogen bonding.

In 2003, the reaction of indoles with aldehydes and ketones under XB catalysis was reported by Bandgar and his research group utilizing I_2_ as the catalyst and acetonitrile as the optimum solvent (Scheme 6) [90]. The scope of this methodology was tested with a variety of substituted aliphatic, aromatic or heterocyclic aldehydes and ketones, affording excellent results. Product conversion rates ranged from 81%, for the less reactive ketones, to 100% for activated aromatic aldehydes bearing electron-withdrawing substituents. The reaction mechanism is based on the activation of the carbonyl group by molecular I_2_, through the formation of a halogen bond, which lowers the LUMO of the carbonyl moiety, increasing its electrophilicity, and thus allowing the addition of the indole group (Scheme 7). The employment of this inexpensive and easily available catalyst under mild reaction conditions, in very short reaction times (<1 min) and with a vast substrate scope, render this protocol practical and economical (Scheme 6) [90].

**Scheme 8:**
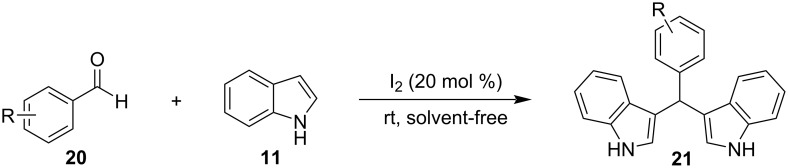
Synthesis of bis(indolyl)methanes using I_2_, introduced by Ji.

In 2004, the Ji group developed a simple and convenient protocol for the synthesis of BIMs, using a catalytic amount of I_2_ under solvent-free conditions at room temperature (Scheme 8) [91]. The differentiating factor of this technique is the employment of solid grind, which avoids the need for a reaction medium, while also utilizing the same amount of I_2_ (20 mol %) compared to Bandgar’s approach, while achieving product yields of 72–90%. Nonetheless, the slower reaction rates (7–10 min) and the limitation of using only aromatic aldehydes, limited the substrate scope and held back more widespread applications of this methodology [91].

**Scheme 9:**
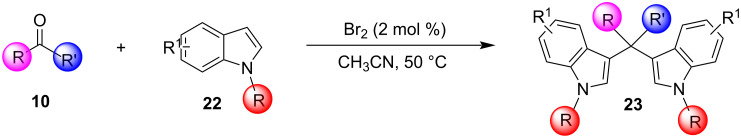
Synthesis of bis(indolyl)methanes using Br_2_ in CH_3_CN.

In 2014, Liang et al. demonstrated an unexpected Br_2_-catalyzed synthesis of BIMs from indole and carbonyl compounds in water (Scheme 9) [92]. First, the reaction took place in acetonitrile with a low catalyst loading (2 mol %), proving sufficient to achieve optimal product yields of up to 98% after just 1 minute, when the reaction mixture was heated at 50 °C. The generality of this approach was also excellent with substituted ketones, aldehydes and indoles, all forming the respective BIMs in yields ranging from 65–98% with ketones displaying the lowest reactivity, requiring up to 12 h for reaction completion. Thus, this protocol is associated with low catalyst loading, extremely high efficiency and broad substrate scope and the possibility of use of both organic solvents or water, with the drawback however, of the employment of conventional heating [92].

**Scheme 10:**
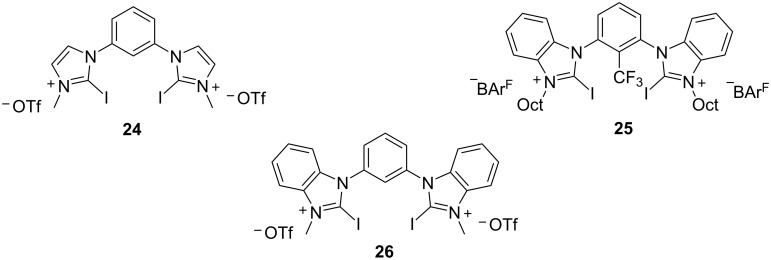
Βidentate halogen-bond donors.

In 2019, Toy et al. proposed an alternative approach for the Friedel–Crafts reaction of aldehydes and ketones with indole [93–94]. Bidentate halogen-bond donors are efficient catalysts, since they can form two halogen bonds with each substrate, instead of just one. Thus, compounds **24**, **25** and **26** were screened for their catalytic activity with **26** emerging as the optimum choice (Scheme 10).

**Scheme 11:**
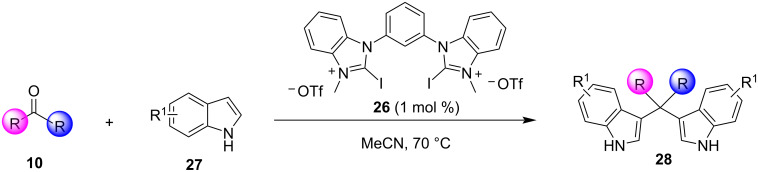
Synthesis of bis(indolyl)methanes using bidentate halogen-bond donor **26**.

**Scheme 12:**
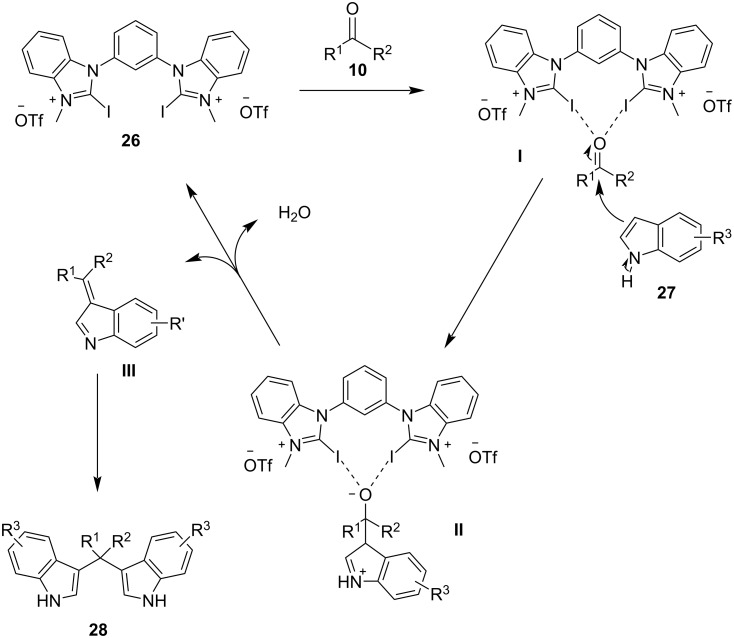
Proposed reaction mechanism.

With catalyst **26** prepared, its use was then studied as a halogen-bond donor in the catalytic synthesis of **28** (Scheme 11) [93–94]. Having identified the optimum reaction conditions, the general applicability was studied by reacting various indoles with a range of aldehydes and ketones to produce a wide range of bis(indolyl)methanes **28** in good to excellent yields (62–93%) [93–94]. Regarding the mechanism of action of this methodology, two halogen bonds are formed between the bidentate halogen-bond donor **26** and the oxygen of the carbonyl group (Scheme 12). This increases the electrophilicity of the carbonyl compound, even more efficiently than the aforementioned catalysts, allowing for the nucleophilic attack of indole as seen in Scheme 12. The following steps are identical to the proposed general catalytic pathway for Lewis acids (Scheme 2). However, this approach did not address the need for conventional heating (70 °C), while also requiring longer reaction times of up to 72 h for sterically hindered substrates [93–94].

**Scheme 13:**
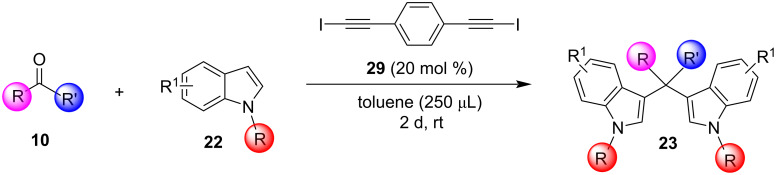
Synthesis of bis(indolyl)methanes using iodoalkyne as catalyst.

**Scheme 14:**
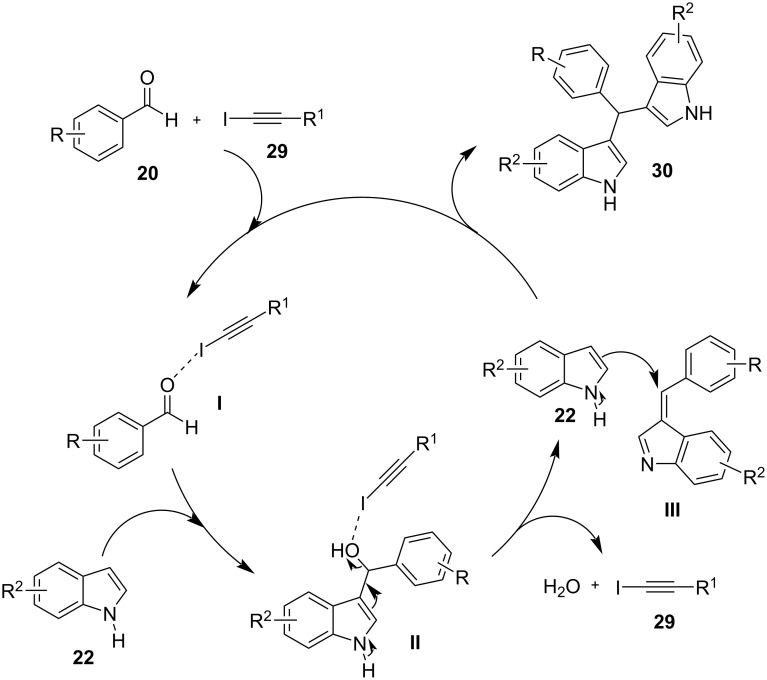
Proposed reaction mechanism.

A novel approach for the formation of BIMs was described, in 2020, by Herrera and co-workers, who utilized iodoalkynes as a simple halogen bond-based organocatalyst (Scheme 13) [95]. Haloalkynes have the ability to form strong, directional and selective halogen bonds, which makes them a good choice for the synthesis of BIMs [95]. Several substituted carbonyl compounds, as well as indoles, were screened in the optimum reaction conditions as seen in Scheme 13 to prove the generality of this protocol. Non-activated aldehydes (not bearing an electron-withdrawing group) and heteroaromatic aldehydes showed a lower reactivity and 30 mol % of catalyst **29** was required to achieve high yields (81–95%). In contrast, activated or aliphatic aldehydes afforded excellent yields (85–98%) in a more facile manner. The reaction mechanism is similar to other halogen-bond donor catalysts (Scheme 14). While the broad substrate scope is a crucial benefit of this approach, the use of a toxic solvent and the slow reaction rates were some of the drawbacks that would need to be addressed.

The most recent application of halogen bonding in the synthesis of BIMs was introduced in 2023 by Galathri et al., who employed an *N*-heterocyclic iod(az)olium salt as the monodentate catalyst [96]. This approach utilized water as the reaction solvent and employed a low catalyst loading of just 0.5 mol %, while providing satisfying yields (60–96%) in just 1 hour. The employment of a green aqueous medium, the mild reaction conditions and the relatively broad substrate scope are some of the benefits that render this protocol more efficient than previous halogen-bonding methodologies [96].

#### Nanocatalysis

Nanocatalysis has emerged over the last decades as a sustainable and green field of organic catalysis that offers unparalleled opportunities for chemical transformations that were previously deemed unfeasible. The use of nanoparticles, compounds with a cross section of less than 100 nm, exhibit various benefits, such as tailoring the scaffold of the catalyst, the recyclability of the nanocompounds, as well as the elevated catalytic activity offered. These benefits stem from their high surface area, that provides more active sites for the reactants to absorb into and collide with one another. With the new avenues offered by the advent of nanocatalysis, it did not take long for its application in the Friedel–Crafts arylation of indoles with aldehydes, since the development of more resource-efficient catalytic pathways for the synthesis of BIMs had received great interest from the scientific world [97–98].

In 2008, the first application of nanocatalysis for the synthesis of BIMs was introduced by Shailaja and her research group, utilizing a ceria/vinylpyridine nanocomposite as the catalyst [99]. After repeated studies and experiments on the reaction between indole (**11**) and benzaldehyde (**31**), methanol emerged as the optimum solvent with the isolated product yield approaching 98%, after 1 hour, when 69 mg of the nanocomposite were added to the reaction mixture [99]. The recovery of the nanocatalyst was feasible by simple filtration and it was found that its catalytic efficiency would not diminish even after 3 cycles [99]. Several substituted indoles, aldehydes and ketones reacted in good yields (74–98%), with ketones requiring longer reaction rates of 3 hours, due to their lower reactivity. The electron-donating or withdrawing effects of the substituents of the benzene ring of the carbonyl groups did not affect this protocol, rendering its generality superior to many traditional approaches. However, the high catalyst loading raised some concerns over the environmental impact of this methodology and left room for improvements for newer approaches [99].

In 2009, Rahimizadeh et al. proposed the use of the nanometal oxide TiO_2,_ which was already reported as an effective nanocatalyst for the promotion of various organic transformations [100]. TiO_2_ nanoparticles are non-toxic, inexpensive and reusable compounds that are synthesized through a sol–gel method. This method involves gradually adding titanium tetra-*n*-butoxide to a solution of deionized water in ethanol and calcinating it to form the desired nanocompounds. 10 mol % of the nano-TiO_2_ heated at 80 °C, provided an optimum yield of 95% for the reaction of indole with benzaldehyde, under solvent-free conditions after just 3 minutes [100]. With the optimum reaction conditions in hand, both aromatic and aliphatic aldehydes reached promising conversion rates of formed BIMs of 77–95%. Sterically hindered substituted aldehydes exhibited longer reaction times, while substituted indoles also showed no issue, reaching yields of 85% after 20 minutes of stirring. The nano-TiO_2_ catalyst was easily recovered by centrifugation, where it could be reused up to four times, without any reductions in product conversion. What holds back the efficiency of this nanocatalytic protocol is the application of conventional heating, as well as the use of a metal oxide at a significant quantity, which can be a water and soil pollutant [100].

**Scheme 15:**
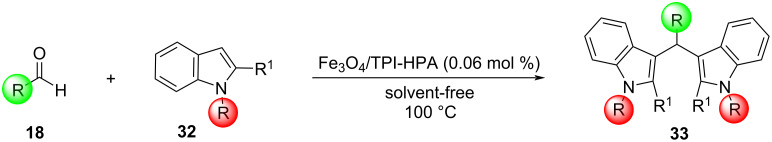
Optimized reaction conditions used by Ramshini.

**Scheme 16:**
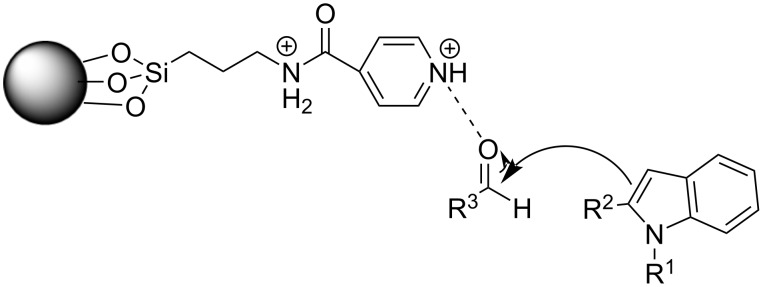
Activation of the carbonyl group by HPA/TPI-Fe_3_O_4._

In 2013, Ramshini and his research group employed H_5_PW_10_V_2_O_40_/pyridino-Fe_3_O_4_ (HPA/TPI-Fe_3_O_4_) as a magnetic nanocatalyst, based on the extensive reported literature about the catalytic properties of Fe_3_O_4_ nanoparticles (Scheme 15) [101–102]. This organic–inorganic hybrid material was synthesized by the immobilization of the dodecatungstovanadophosphoric acid (HPA) on TPI-Fe_3_O_4_ with *N*-[3-(triethoxysilyl)propyl]isonicotinamide (TPI), acting as the linker for the heterogeneous catalyst, while preventing leeching, since the HPA is anchored on the inert and porous Fe_3_O_4_. After obtaining the magnetic nanocompound, different reaction conditions were tested, where it was surprisingly discovered that in the absence of any solvent, higher product yields (96%) were attained with a catalyst loading of just 0.06 mol % in just 25 minutes [101–102]. Furthermore, the nanocatalyst could be easily retrieved by applying a magnetic field on the reaction mixture, where it could be reused for at least 8 cycles, before there was a noticeable drop in effectiveness. This boosted the sustainability of this protocol significantly, with the drawback of needing conventional heating at 100 °C for the composite material to activate the reacting carbonyl group by lowering its LUMO orbital, rendering it a more potent electrophile so that the nucleophilic indole can attack it (Scheme 16) [101–102].

Both aliphatic and aromatic aldehydes faced no problems, reaching yields ranging from 65–98% of formed products with substituted indoles also showing exceptional reactivity, albeit no ketones were reported forming the respective BIMs, limiting the substrate scope. Therefore, the solvent-free conditions of the reaction, the low catalyst loading and the ability of its retrieval rendered this methodology a green alternative to traditional protocols, however, the costly requirement of conventional heating hinders industrial applications.

At the same time that Ramshini published her approach, Jahanshahi also introduced nano *n*-propylsulfonated γ-Fe_2_O_3_ (NPS-γ-Fe_2_O_3_), which constitutes a magnetically recyclable heterogeneous catalyst that works in the exact same manner as HPA/TPI-Fe_3_O_4_ [103]. Some small differences between the two methods were the ability of ketones to form the respective BIMs, when NPS-γ-Fe_2_O_3_ was employed in contrast to HPA/TPI-Fe_3_O_4_, while also using a milder heating at 80 °C. However, Ramshini’s protocol had shorter reaction times of 25 minutes instead of 1 to 5 hours and also the catalyst loading was significantly decreased at 0.06 mol %, instead of the 0.5 mol % needed for NPS-γ-Fe_2_O_3_. Other than these differences the mechanism of action, the received yields, the absence of solvent and the recoverability of the nanocompound were identical between these two studies [103].

In the same year, another solvent-free approach was introduced by Chen et al. that utilized sulfated zirconia nanoparticles to conduct the Friedel–Crafts reaction between indoles and aldehydes [104]. In an effort to synthesize the sulfated zirconia nanocompounds, a new two-step precipitation method was developed. The first step involved the employment of zirconium oxychloride and its precipitation with ammonium hydroxide. In the second step, the formed zirconium hydroxide undergoes a sulfate impregnation, utilizing sulfuric acid in the presence of polyvinylpyrrolidone (PVP) as the surfactant, to prevent particle agglomeration, leading to the formation of the desired sulfated zirconia nanoparticles after a last calcination step. After obtaining the nanocompound, its catalytic activity was evaluated for several organic transformations, including the synthesis of BIMs. Diindolylphenylmethane (DIM) was obtained in a yield of 97%, when 30 mol % of the nanocatalyst was added after 24 hours of reaction time in solvent-free and room temperature conditions. In these optimized conditions, several aromatic aldehydes and indoles formed the respective BIMs in excellent yields (84–95%), without any noteworthy differences between the various carbonyl substrates. Indoles, substituted at the 2’ position, were much more reactive, leading to isolated product yields of around 95% just after 6 h. This protocol addressed the issue of conventional heating that the previous methodologies employed, while also maintaining the solvent-free character of the reaction, with the disadvantage of longer reaction times, a high amount of nanocatalyst utilized and a more restricted substrate scope [104].

**Scheme 17:**
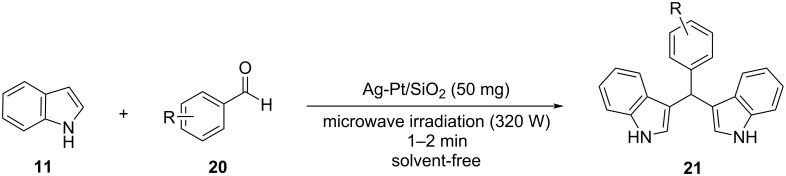
Synthesis of BIMs in the presence of nanoAg-Pt/SiO_2_-doped silicate_._

Karthikeyan and his research team synthesized Ag-Pt nanoparticles suspended in silicate, with the sol–gel method analyzed before, to test their effectiveness in the synthesis of BIMs, since they had already been applied as nanocatalysts in various oxidation and hydrogenation reactions [105]. NanoAg-Pt doped silicate constitutes an efficient and recyclable catalyst that can be reused without a notable loss in catalytic activity (Scheme 17). Wanting to avoid the use of conventional heating, Karthikeyan and his co-workers turned to microwave irradiation (320 W), which resulted in rapid reaction rates with isolated product yield reaching 92% in just 1 minute, while also maintaining solvent-free conditions. Different amounts of silicate were also examined with 50 mg of the nanocompound, providing the most satisfying results, while in the absence of the nanocatalyst, no product was formed, highlighting its importance in this protocol. A wide range of aromatic aldehydes were employed without encountering any difficulties in reactivity and obtaining conversion rates ranging from 78–95% in a maximum of two minutes for substrates containing more than two electron-donating substituents on the benzene ring. However, no ketones or aliphatic substrates managed to exhibit sufficient reactivity. The nanoAg-Pt silicate could be easily recovered by a simple filtration with methanol, allowing it to be reused for several catalytic cycles, before it was rendered inactive. Thus, Karthikeyan’s approach implemented faster reaction rates, while avoiding conventional heating with the application of microwave-assisted irradiation in solvent-free conditions with the drawback of limiting the generality of his methodology [105].

In 2014, Chabukswar and his research group explored the activity of cadmium sulfide (CdS) nanotubes as heterogeneous nanocatalysts for the electrophilic condensation between aldehydes and indoles [106]. The nanorods were obtained with a solvothermal technique, where thiourea and cadmium nitrate were mixed in ethylenediamine for 10 minutes and subsequently heated at 200 °C for 12 hours. After being centrifuged, washed with water and grinded in a mortar, they were ready to be tested in the model reaction of benzaldehyde with indole. The optimum product yield was reached with a catalyst amount of 5 mol %, as a further increase did not show any further enhancement, in reflux temperature (65 °C) conditions, with methanol emerging as the superior medium over other polar solvents. A wide range of aromatic aldehydes containing both electron-withdrawing and electron-donating substituents were employed with the former, improving the conversion rate of the formed BIM product to 90–95%, compared to the 85–87% of the latter after 15 minutes of reflux. The nanotube catalyst was retrieved with a simple filtration, utilizing ethyl acetate and was reused for four synthetic cycles, before there was a decrease in catalytic activity, which was attributed to deactivation of active sites of the nanorod. Once again, this method provided short reaction times with an efficiently recovered catalyst with the handicap of the necessity of conventional heating and an unfavorable solvent [106].

**Scheme 18:**
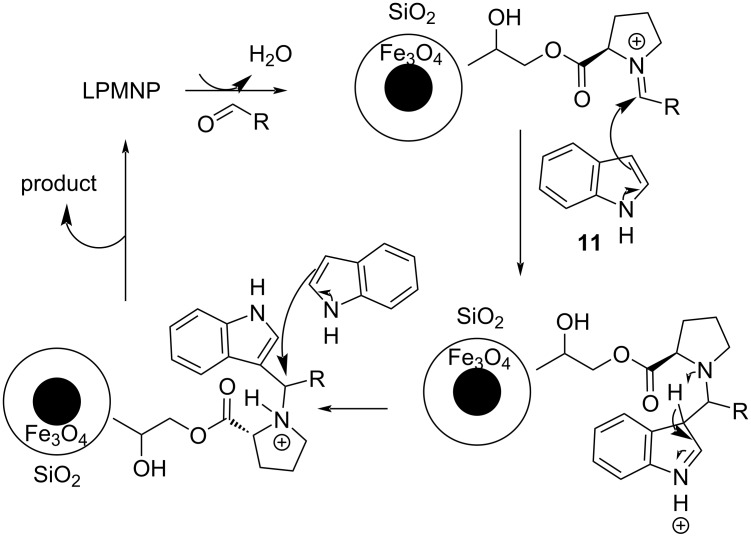
Mechanism of action proposed by Khalafi-Nezhad et al_._

At the same time, Khalafi-Nezhad et al. developed unique ʟ-proline-modified magnetic nanoparticles (LPMNPs) that combine organocatalytic protocols with nanocatalysis, which enhances the surface-to-volume ratio of the catalyst opening up new possibilities [107]. The ʟ-proline molecules were anchored on a Fe_3_O_4_@SiO_2_ nanoparticle, which was already known for its facile recyclability, with the silica layer preventing the Fe_3_O_4_ from aggregation. The LPMNPs managed to provide impressive yields, while employing an amount of 2.5 mol % in water, with the help of conventional heating at 50 °C, which facilitated the organocatalytic process that required 1 to 1.5 hours to reach completion. Various aromatic aldehydes reached conversion rates of 80–94% with electron-donating substituents, enhancing the reactivity of the reagents, compared to their electron-withdrawing counterparts that afforded lower isolated product yields. Subsequently, the catalyst reusability was also evaluated, where it was observed that even after 8 cycles without any treatment the morphology of the nanotubes remained the same, without leaching of considerable amounts of ʟ-proline into the reaction mixture. The mechanism of action involves the activation of the aldehyde by the grafted ʟ-proline on the surface of the magnetic nanoparticle via iminium formation as seen in Scheme 18, allowing the indole to attack the formed double bond and initiate the Michael-type mechanism [107].

In an effort to replace the widespread use of nano-iron oxides, which present issues, such as aggregation, and requires large amounts of capping agents to combat, Pratihar and his research group offered the alternative of iron oxalates for the nanocatalysis of BIMs, which, after thermal decomposition form Fe(ox)-Fe_3_O_4_ oxides [108]. Metal oxalates in general are more stable nanoparticles than their oxide counterparts, while retaining their magnetic and catalytic properties preventing the aforementioned drawbacks. Compared to Ramshini’s protocol for the synthesis of BIMs however, a higher catalytic loading was required (5 mol %) and water was added as the solvent, with both approaches also requiring conventional heating of the resulting mixture at 100 °C. Reaction rates were also slightly lower, requiring 1 hour for aromatic aldehydes containing electron-withdrawing groups and upwards of 4 hours for electron-donating substituents compared to the 25 minute Ramshini’s protocol with the additional holdback of the lack of reactivity of aliphatic aldehydes and substituted indoles. The recyclability of the nanocompound was satisfactory, since it could be reused for at least 5 times, before a slight drop in conversion rates was observed. However, due to the choice of water as the solvent, insoluble product clamps were formed, which led to a more complex recovery of the catalyst as firstly the water needed to be removed by decantation and next acetone was added so that the BIMs were dissolved and the nanoparticles could be retrieved with the use of a tiny magnet. All in all, while the iron oxalates combatted some disadvantages of the use of iron oxides, the catalytic approach presented had handicaps that held back its broader applications [108].

**Scheme 19:**
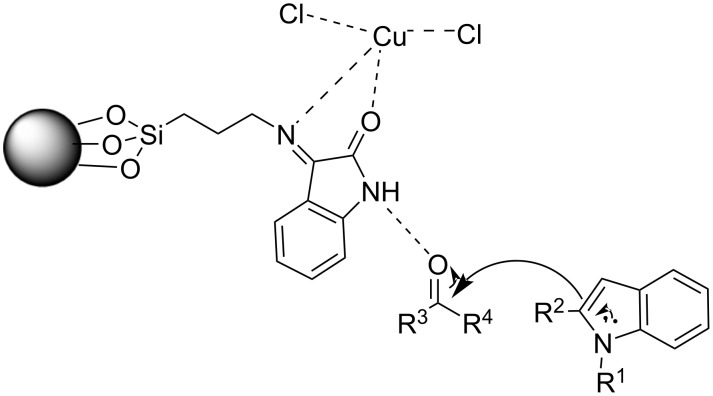
Activation of the carbonyl group by the Cu–isatin Schiff base complex.

In 2016, Sobhani et al. expanded on the use of iron oxide as an effective magnetic nanoparticle by creating a Cu–isatin Schiff base complex supported on nano-iron oxide compounds for the synthesis of BIMs [109–110]. These compounds were synthesized through the reaction between the amino-functionalized modified magnetic nanoparticles (MNPs), which were obtained by a sol–gel methodology, and isatin, producing isatin Schiff base-γ-Fe_2_O_3_, which was subsequently dissolved in methanol with CuCl_2_, yielding the desired Cu–isatin Schiff base complex. The optimum reaction conditions between benzaldehyde and indole were obtained at a nanocatalyst loading of only 0.25 mol % after 2 hours of heating at 80 °C with water as the medium. In the absence of the Cu–isatin Schiff base complex, only trace amounts of product were observed, which further underlined the importance of its presence in the reaction mixture. The biggest benefit, however, of this approach is its generality, since all types of substituted carbonyl compounds and indoles, provided conversion rates ranging from 80–98%, with aromatic aldehydes bearing electron-donating substituents requiring the longest reaction times that approached 5 hours. The recoverability and reusability of the nanocomplex were similar to that of other nano-iron oxide compounds, since it could be reapplied in the reaction mixture for up to 8 cycles before signs of deactivation were observed. The mechanism of action was also identical with the Cu–isatin Schiff base complex activation of the carbonyl group, facilitating the electrophilic addition of indole (Scheme 19). The main drawback of the method, however, is the necessity of conventional heating and solvent compared to other methodologies [109–110].

At the same time, Yavari and his research group also studied the use of hexamine, in place of NPS-γ-Fe_2_O_3_, immobilized on Fe_3_O_4_ and coated with SiO_2_ [111]. This protocol, while employing the identical optimum reaction conditions with Ramshini’s method, managed to reduce the reaction times to 10 minutes with 10 mg of the catalyst and could also facilitate the reaction of ketones. This improved upon the previously known nano-iron oxide methodologies without sacrificing their already satisfying product yields, the recyclability of the nanocatalyst or their environmentally benign aspects, such as the solvent-free conditions without, however, addressing the need for conventional heating that still presented an issue [111].

**Scheme 20:**
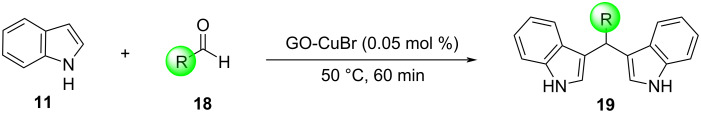
Optimum reaction conditions published by Jain.

The same year, Jain and her research team opted out of the use of silica as the support for their nanocompounds, instead picking graphene oxide (GO), due to its unique morphology and high chemical stability (Scheme 20) [112]. The graphene oxide was decorated with CuBr nanoparticles, which had already shown great catalytic potential, forming the GO–CuBr complex that was utilized for the synthesis of BIMs. The researchers were delighted to discover that 0.05 mol % of the nanocompound were sufficient to promote the reaction between benzaldehyde and indole at 50 °C affording the product in 92% yield after 1 hour. In the absence of the catalyst, a trace amount of product was formed, while reducing the amount further resulted in a lower conversion. Next, the generality of the method was explored by employing several aliphatic and aromatic aldehydes, as well as substituted indoles, in various positions of the benzene ring. *N-*Substituted indoles displayed the lowest reactivity, providing yields around 75%, while aldehydes had no difficulties reaching conversion rates of 87–92%. However, no ketones were reported in the substrate scope. The nanocomplex was retrieved following reaction completion by centrifugation and could be reapplied for up to 6 cycles, before a noticeable drop in catalytic activity occurred. Thus, a solvent-free approach was reported with the lowest amount of nanocatalyst applied, compared to previous methodologies. However, conventional heating was still needed and not all carbonyl compounds managed to form the respective BIMs in the present conditions [112].

The introduction of graphene oxide would be expanded upon and enhanced by Masram and his research group a year later [113], who implemented La_2_O_3_ nanoparticles in place of the CuBr MNPs that Jain used, in solvent-free conditions. The La_2_O_3_/GO complex provided much faster reaction rates (25–60 min) and improved product conversion rates (88–97%) without the necessity for heating that held back the GO–CuBr complex. These benefits came, however, at the cost of a higher catalyst loading of 5 mol % and worse reusability, since after just 2 reaction cycles the product yields dropped by 10%, providing an interesting alternative to the employment of GO–CuBr without overshadowing its applications [113].

Shirini et al. utilized the polymer poly(4-vinylpyridine) (P4VPy) for the immobilization of nanoparticles in lieu of silica, due to the strong affinity of the pyridyl group towards metals and its ability to undergo hydrogen bonding with polar species [114]. The nanoparticle of choice was CuO, which had already displayed several varied catalytic applications, so the P4VPy–CuO nanoparticles were synthesized through an ultrasound irradiation protocol and tested in the reaction between *p*-chlorobenzaldehyde and indole. An amount of 20 mg of the P4VPy–CuO nanocompound at 80 °C proved sufficient, under solvent-free conditions, at providing an optimum isolated product yield of 92% after 5 minutes. Various indole derivatives and carbonyl compounds were explored, where it was found that ketones were less reactive, due to their lower electrophilicity and required upwards of 2 hours to reach completion, while aldehydes containing electron-withdrawing substituents required just 4 minutes. The nanopolymer was promptly filtered and reused for up to 3 cycles with satisfying activity. BiVO_4_ nanoparticles were introduced by Lati and his research group for the synthesis of BIMs applying the same catalyst-free conditions as P4VPy–CuO, while employing 30 mg of the nano-BiVO_4_ compound with the added benefit of slightly improved product yields, ranging from 70 to 98% after 10–80 minutes [115–116]. The need for conventional heating, the reaction rates, the mechanism of action and the recyclability of the nanocatalyst used were comparable in both approaches with the only differences being the choice and amount of the nanocomplex added and certain product conversion rates. Inspired by this technique, in 2019 Boroujeni et al. immobilized carbon nanotubes on sulfonated polyacrylamide creating polymer/carbon nanotube composites, which could also be employed in the synthesis of BIMs [115–116]. This methodology used a catalyst loading of 5 mol % under reflux conditions (85 °C) in acetonitrile for 30–40 minutes with product yields ranging from 90 to 97%. Despite the drawback of the use of a solvent this protocol succeeds in incorporating aliphatic carbonyl compounds in the substrate scope and an effective nanocatalyst that can be retrieved and reused for 8 cycles without a drop in catalytic activity [114–116].

**Scheme 21:**
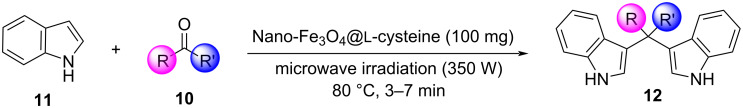
Organocatalytic protocol utilizing nanoparticles introduced by Bankar.

In 2018, Bankar inspired by the use of ʟ-proline-modified magnetic nanoparticles reported by Khalafi-Nezhad et al., synthesized nano-Fe_3_O_4_@ʟ-cysteine for the green synthesis of BIMs, employing microwave irradiation to avoid long reaction times [117]. A mixture of the reagents and 100 mg of nano-Fe_3_O_4_@ʟ-cysteine per 1 equiv of the carbonyl compound, was exposed to 350 W of microwave irradiation, at an internal temperature of 80 °C, at solvent-free conditions, for 3 to 7 minutes, providing optimum yields for aromatic aldehydes and ketones. Isolated product yields approached 83–93%, even after 10 reaction cycles with the nano-Fe_3_O_4_@ʟ-cysteine particles being magnetically recoverable in a facile manner. The reaction could also be realized with conventional heating at 80 °C, however, the reaction rates were more than 20 times lower, and ketones displayed unappealing conversion rates. The mechanism of action follows the one suggested by Khalafi-Nezhad et al. for LPMNPs, where activation of the carbonyl compound by the nanocatalyst initiates the indole attack (Scheme 21) [117].

**Scheme 22:**
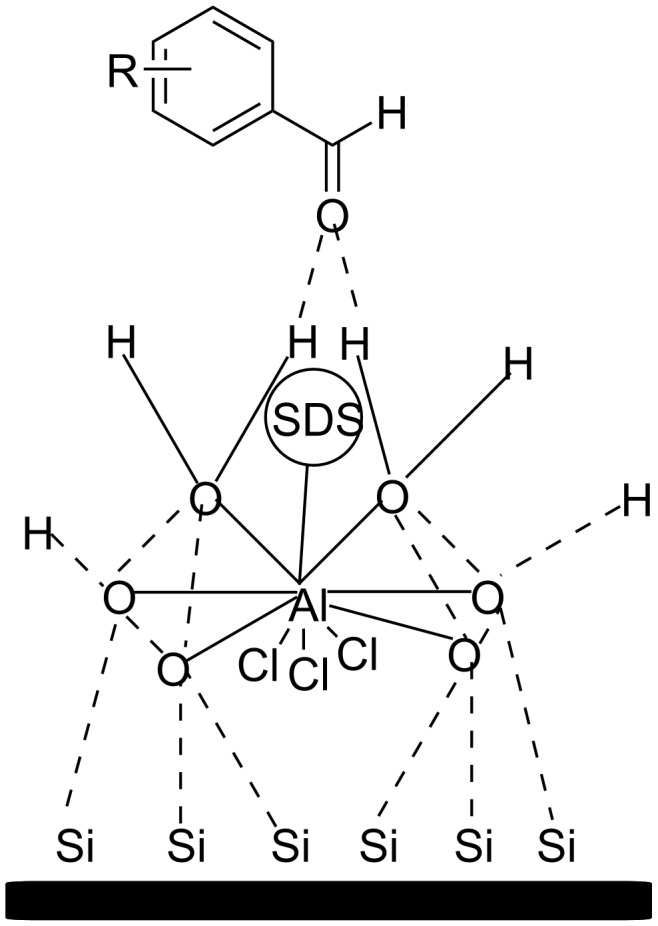
Activation of the carbonyl group by the AlCl_3_·6H_2_O-SDS-SiO_2_ complex.

A year later, Chen and his research team created Lewis acid-surfactant-SiO_2_-combined (LASSC) nanocatalysts by combining Lewis acid surfactants with SiO_2_, thus, eliminating the drawback of poor recyclability exhibited by the surfactants [118]. Various different Lewis acid surfactants were screened for their catalytic activity in the synthesis of BIMs with the combination of AlCl_3_∙6H_2_O as the Lewis acid, sodium dodecyl sulfate (SDS) as the surfactant and silica as the carrier providing the most promising results. Solid grinding was also employed, removing the need for a solvent and accelerating the reaction rate to 20 minutes and product yields to 99% without the need for conventional heating. Replacement of the Lewis acid or the silica greatly diminished the conversion rates of BIMs, while replacing SDS with different surfactants had no effect on the reaction profile. Even though a large amount of 0.5 g of the LASSC nanocatalyst was employed, it displayed impressive stability, since it could be retrieved and reused upwards of 11 times without any structural changes, making up for the high catalyst loading. This protocol was applied to reactions of various aromatic aldehydes and indoles with excellent yields, where it was observed, as in most other cases, that substrates with electron-withdrawing groups achieved better yields in shorter times, compared to their electron-donating counterparts. The mechanism of action involves the Al atom, which is located at the center of the regular octahedron (Scheme 22), forming hydrogen bonds with the carbonyl group, facilitating the electrophilic reaction with indole by lowering the LUMO orbital of the C=O bond. Thus, an innovative approach on nanocatalysis was introduced, incorporating solid grinding in catalyst-free conditions with the challenge of a high catalyst loading and lack of aliphatic aldehydes or ketones being utilized as substrates for the formation of their respective BIMs [118].

In 2021, this methodology was reiterated by Wu et al., who conducted the reaction in the exact same conditions, utilizing the ball milling technique, instead of solid grinding, which slightly reduced reaction times and the catalyst loading necessary from 500 mg to 300 mg [119]. The substrate scope was also expanded upon, including aliphatic substrates and enhancing the possible LASSC nanocatalyst applications further [120–121].

**Scheme 23:**
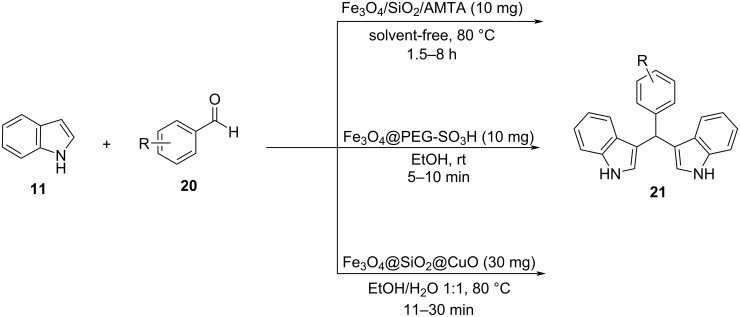
Optimal reaction conditions for the aforementioned nano-Fe_3_O_4_ based catalysts.

In the following years between 2020 and 2022, focus was still on the development of nanocatalysts based on iron oxides applied for the synthesis of BIMs, since they were easy to make, cheap and easily recoverable, while also displaying high catalytic activities. Specifically, three protocols utilizing Fe_3_O_4_ nanoparticles were introduced in this time frame, with the first being published in 2020 by Shafiei and her research group [120–121]. They synthesized 3-amino-5-mercapto-1,2,4-triazole (AMTA)-functionalized Fe_3_O_4_ nanoparticles coated on silica, based on Khalafi-Nezhad’s approach. Optimum catalytic activity was attained when adding 10 mg of the nanocomplex to the reaction mixture, in solvent-free conditions at 80 °C and after a time period of 1.5–8 hours, depending on the substrate used, with both aromatic and aliphatic aldehydes reaching conversion rates of 98% (Scheme 23). In 2021, a methodology making use of poly(ethylene glycol)-supported Fe_3_O_4_ nanoparticles was reported by Mardani et al., who managed to remove the need for conventional heating. 10 mg of Fe_3_O_4_@PEG-SO_3_H complex were added in ethanol for a time period of 5 to 10 minutes, yielding 88–98% of isolated product with only aromatic aldehydes showing a meaningful reactivity (Scheme 23) [122]. In 2022, Boroujeni et al. employed a Cu(II) complex coated in Fe_3_O_4_@SiO_2_ nanoparticles which worked as an efficient nanocatalyst for the synthesis of BIMs in a catalytic amount of 30 mg at 80 °C [123–124]. A mixture of water and ethanol (1:1) was discovered to be the optimum solvent with reaction times starting from 11 to 30 minutes, while approaching yields ranging from 92% for aliphatic aldehydes to 97% for aromatic aldehydes (Scheme 23). All applications shared the same mechanism of action based on the activation of the carbonyl bond as shown in Scheme 22 with each method presenting new environmental benefits compared to the traditional acid-catalyzed approaches [120–124].

In 2020, Ozturk and his research group developed a novel sulfonic graphitic carbon nitride (g-C_3_N_4_-SO_3_H) as a nanosheet ionic liquid for the one-pot synthesis of BIMs [125]. Graphitic carbon nitride is a non-metal and non-toxic chemically stable nanostructure, which is obtained through a facile pyrolysis process that has not seen wide applications in nanocatalysis despite its numerous benefits. Therefore, Ozturk made it usable by coupling it with an ionic liquid, creating g-C_3_N_4_-SO_3_H nanoparticles, which displayed high catalytic activity for the Friedel–Crafts arylation of indoles with aldehydes. The optimum product yields were achieved with 15 mg of g-C_3_N_4_-SO_3_H (IL) added in a mixture of ethanol and water (3:1) at 70 °C after a period of 1 hour. Several aliphatic and aromatic aldehydes completed the reaction successfully with conversion rates of 82–98%, with the electronic effect of the substituents having no effect on reaction times or yields. The nanostructure could be recovered by simple filtration, where it could be reemployed upwards of 5 times before a drop in catalytic activity was noticed. The low reactivity exhibited by ketones, the use of ethanol as a solvent and the need for conventional heating were some issues that held back the universality of this protocol [125].

**Scheme 24:**
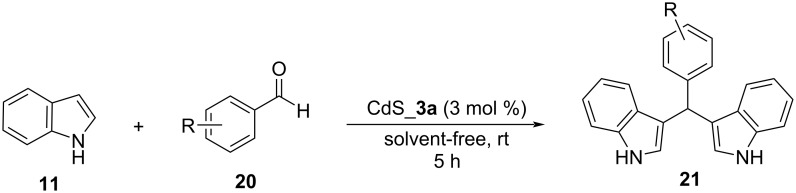
Nanocatalytic protocol proposed by Kaur et al.

More recently, in 2022, Kaur et al. utilized CdS nanostructures which possess good Lewis acidity for the heterogeneous catalysis of various organic transformations including the synthesis of BIMs as shown by Chabukswar and his research group [126]. Kaur et al. synthesized various CdS nanostructures with differences in their morphology and screened them for their catalytic potential, while avoiding the use of surfactants, which were too difficult to remove from the reaction mixture (Scheme 24). CdS_**3a** which was a porous and hollow open interconnected network of CdS nanoparticles emerged as the optimal catalyst structure. This structure was found to promote the reaction between indole and *p-*chlorobenzaldehyde in solvent-free conditions after 5 hours at a catalyst loading of 3 mol %. Several aromatic aldehydes were also tested in these conditions with substrates bearing electron-withdrawing groups, displaying much higher conversion rates of 60–99% than electron-donating groups, whose reactivity was low, with conversion rates ranging from 30 to 45%. The CdS_**3a** nanoparticles were easily retrievable through the application of centrifugation and could be reused three times, before an observable change in their catalytic activity occurred. While the mild reaction conditions and the lack of solvent or conventional heating aided the environmental impact of this approach, the limited substrate scope of just aromatic aldehydes bearing electron-withdrawing groups hindered its application and generality considerably leaving room for further improvements for the employment of CdS nanoparticles. However, the employment of CdS nanoparticles is not considered sustainable, despite their recyclability, due to their toxicity and carcinogenic properties (Scheme 24) [126].

#### Microwaves

With the increased interest in the development of greener catalytic protocols targeting the decrease in chemical waste, microwave irradiation (MW) has emerged as a popular heating technique for the synthesis of BIMs and many other organic compounds, presenting appealing pharmacological properties. Under microwave irradiation, chemical reactions are accelerated by the absorption of microwaves by polar organic molecules, which renders reactions that are not catalyzed with conventional heating possible. This was made apparent in Karthikeyan’s and Bankar’s nanocatalysis protocols for the synthesis of BIMs, who incorporated MW, which led to an impressive surge in reaction rates [105–117]. The prominent benefits of the microwave approach over traditional protocols, are low energy consumption, accelerated reaction rates, avoiding the use of toxic organic solvents and ease of regulation of the reaction parameters [127–128].

**Scheme 25:**
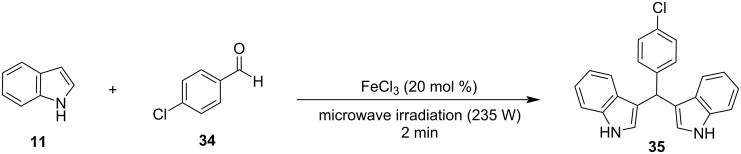
Microwave approach introduced by Yuan.

**Scheme 26:**
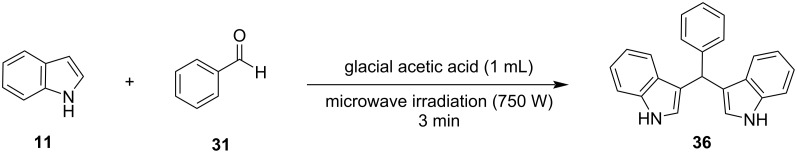
Microwave approach introduced by Zahran et al.

The first solvent-free methodology based on the microwave approach for the Friedel–Crafts reaction of aldehydes with indoles was presented by Yuan and his research team in 2004 [129]. They tested a range of Lewis acids for their catalytic activity in the electrophilic substitution with indole. It was found that FeCl_3_ at 20 mol % catalyst loading provided the best yield (93%) for the reaction of *p*-chlorobenzaldehyde with indole, while also having a reaction time of just 2 minutes (Scheme 25). The best option for the irradiation power was found to be 235 W, since when it was weaker, the yield of the product was lower than 93% and when it exceeded 235 W, the acid was deactivated, and no product was formed. After the reaction conditions were optimized, many substituted aldehydes were tested, and it was discovered that aromatic substrates gave the products in satisfying yields (72–93%). However, aliphatic carbonyl compounds were not able to form the corresponding BIMs with the use of this protocol [129]. In the next years, there were more microwave approaches published with the following one emerging in 2008 by Zahran et al., who used glacial acetic acid (1 mL), instead of FeCl_3_, and an irradiation power of 750 W (Scheme 26) [128]. The reaction mixture was subjected to successive 30 second irradiation periods to prevent deactivation of the catalyst. The reaction reached completion in 1 to 60 minutes depending on the substrate, with aliphatic aldehydes once again displaying low yields and reaction rates. The reactions were also carried out in acetic acid with conventional heating, where it was observed that compared to the microwave approach, the isolated product yields were lower and the reaction rates more than 10 times slower. Another benefit of this methodology is the facile work-up of the formed products for their isolation, however, the high catalyst loading of the glacial acetic acid and low tolerance for aliphatic substrates limited some of its applications [128].

**Scheme 27:**
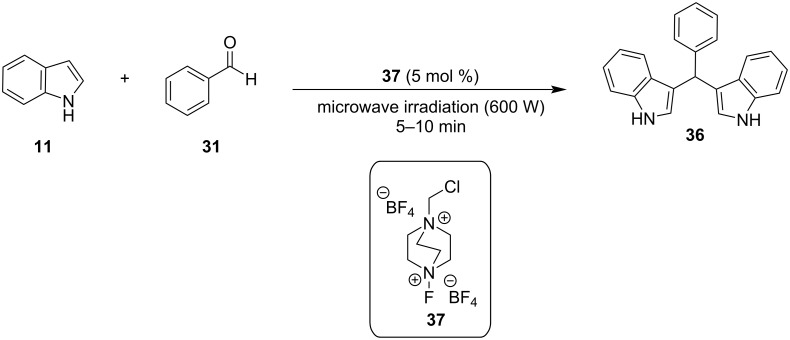
Microwave irradiation protocol introduced by Bindu.

A year later, in 2009, Bindu and her research group introduced Selectfluor (**37**), which is an electrophilic fluorinating agent that can act as a Lewis acid for the synthesis of BIMs with the use of microwave irradiation (600 W) (Scheme 27) [130]. This protocol utilized 5 mol % of catalyst loading, which is an advantage over previous methodologies and reaction times of 5 to 10 minutes to reach optimal yields of isolated products (85–96%). However, just like previously the drawback presented is the low reactivity of aliphatic products, which present a challenge for microwave-based catalysis [130].

**Scheme 28:**
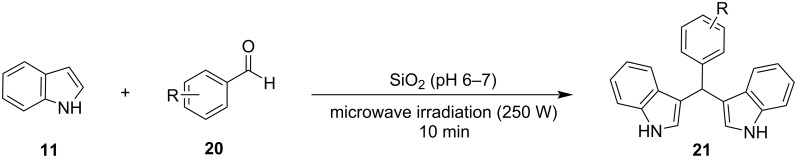
Silica-supported microwave irradiation protocol.

In 2014 Gu, and his research group reported an interesting application of SiO_2_, which acts as a solid support under microwave irradiation and is slightly acidic (pH 6–7), rendering the use of another Lewis acid or solvent redundant (Scheme 28) [131]. SiO_2_ was discovered to be the best solid support over other similar compounds like Al_2_O_3_, possibly due to its larger surface area and low gravity, while also being relatively inexpensive and commercially available only facilitated its choice as the optimum choice. The reaction mixture containing SiO_2_ (1.0 g) was irradiated for 10 minutes under 250 W to achieve the best possible yield, since any time exceeding that of 10 minutes lowered the product formation, possibly due to its carbonization. All products from aromatic aldehydes were isolated in satisfying yields ranging from 85–98% with the existence of electron-withdrawing substituents on the benzene ring of the aldehyde leading to higher yields, in contrast to electron-donating substituents. Aliphatic aldehydes once again did not lead to the formation of the desired BIM, however, many BIMs containing isoxazole groups were synthesized with this method which have displayed many interesting medicinal applications [131].

**Scheme 29:**
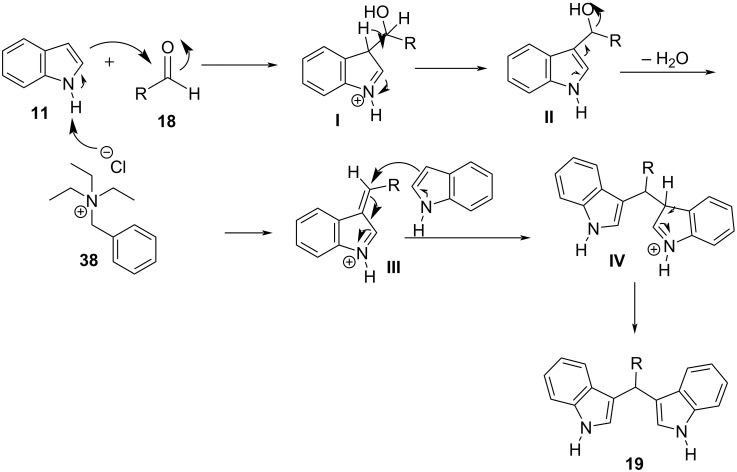
Proposed mechanism for formation of BIM by Nongkhlaw.

A year later, in 2015, Nongkhlaw and his research lab incorporated phase-transfer catalysts into their microwave irradiation protocol to utilize water as the reaction solvent, in order to avoid the possibility of combustion or charring of the reaction mixture [132]. Several different phase-transfer catalysts were tested for their efficacy with benzyltriethylammonium chloride (TEBA, **38**), emerging as the optimum choice, while only needing a catalyst loading of 0.25 mol % to achieve product yields of around 95% at a low irradiation power of 120 W. Reaction times varied depending on the substrates used from 2 to 4 minutes and in contrast to previous protocols, aliphatic aldehydes can also react giving high product yields of 85–98%. Another benefit of the TEBA/water catalytic system is its reusability, since it can be reused for three catalytic cycles before there is a significant decrease in the yield of the product observed. The proposed mechanism of action of TEBA (as seen in Scheme 29) involves its chloride anions, transferring the reactants towards the aqueous phase so they can react leading to the formation of the corresponding BIM using a simple Michael-type mechanism. This protocol can also be used for the synthesis of tetraindolylmethanes, justifying the drawback of the use of a solvent compared to the rest of the microwave-assisted methodologies [132].

**Scheme 30:**
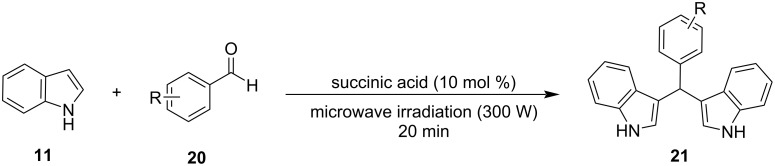
Microwave-assisted synthesis of BIMs catalyzed by succinic acid.

Following Nongkhlaw’s study, in 2017, Rao and his research group introduced a new microwave-assisted catalytic protocol, utilizing water once again as the solvent and succinic acid as the catalyst in place of TEBA (Scheme 30) [133]. Succinic acid provided the most promising results, compared to other naturally derived organic acids, and the fact that it is a safe, commercially available, non-toxic and biodegradable compound made it an appealing choice. The reaction conditions were optimized for the reaction between indole and benzaldehyde, where it was found that the corresponding BIM was formed with a yield of 96%. This result was obtained when the reaction mixture was irradiated under 300 W for 20 minutes with 10 mol % of succinic acid. After optimizing the reaction conditions, the focus shifted towards the substrate efficiency of the succinic acid protocol. Both aliphatic and aromatic aldehydes afforded the respective products in excellent yields (78–96%) although, ketones displayed low reactivity and did not provide any product. Control experiments were also conducted, and it was observed that no product was formed in the absence of succinic acid. At the same time, the product yield only reached 50% after 24 hours without microwave irradiation, highlighting the necessity of both for the synthesis of BIMs. The reaction mechanism is a simple acid-catalyzed Friedel–Crafts bisarylation. The aldehyde is activated by the succinic acid and subsequently undergoes an electrophilic substitution at C-3 and after the loss of water and the addition of the second molecule of indole the corresponding BIM is formed [133].

**Scheme 31:**
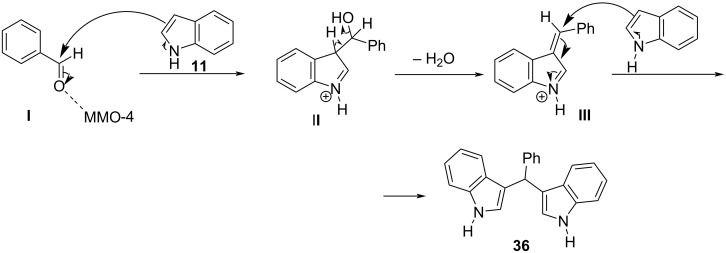
Proposed mechanism of action of MMO-4.

The most recent study for the arylation of aldehydes with indoles under microwave irradiation was published in 2018 by Nguyen et al., who made use of porous metal oxides derived from Cu-Al layered double hydroxide as heterogeneous catalysts [134]. Four different mixed metal oxides (MMOs) and layered double hydroxides (LDHs) were tested with different analogies of Cu-Mg-Al to find the one that displayed the highest catalytic activity with MMO-4 (0:3:1) prevailing over the rest. MMO-4 is the result of the calcination of LDH-4, which leads to the increase of its surface area and explains the difference in catalytic activity despite having the same analogies of Cu-Mg-Al. After finding the most suitable catalyst, the reaction conditions were optimized. It was found that the highest product yields were achieved, when 10 mg of catalyst was used for 10–20 minutes of microwave irradiation at 160 °C and in solvent-free conditions. Both substituted aromatic aldehydes and indoles provided the expected product in the range of 71–98% yield with benzaldehydes substituted at the *para*-position with a halogen, displaying the lowest yields, while indoles and benzaldehydes substituted with strong electron-withdrawing substituents had the highest reactivity. MMO-4 also has the benefit of reusability, since it can be easily retrieved from the reaction mixture and it only starts to lose catalytic activity after the fifth cycle, which makes it a promising choice for the application on industrial processes. The proposed mechanism of action as shown in Scheme 31 begins with MMO-4, increasing the electrophilicity of the carbonyl group by lowering its LUMO orbital and next the reaction follows the steps of a simple Michael addition, like TEBA. The drawback of this method is the use of a metal catalyst, however, the low catalyst loading, the recyclability of the catalyst and the absence of solvent renders this protocol more environmentally benign than other traditional alternatives that utilize metal catalysts [134].

#### Ultrasound

Ultrasound irradiation is an unconventional energy source, compared to conventional heating, that is receiving increased interest for its applications in organic catalysis as a more sustainable and byproduct-free alternative synthetic protocol. Sonochemistry is based on the local depression of liquids, which causes a drop in their vapor pressure, leading to the generation of cavitation bubbles. These cavitation bubbles absorb the energy provided by the generated sound wave, causing them to grow in size until they collapse, resulting in the generation of shock waves or radicals that can initiate various chemical processes. Ultrasound irradiation enhances product selectivity, while having a low energy consumption overall and it activates new mechanical pathways for known reactions [135].

The first practical synthesis of BIMs under ultrasound irradiation was introduced by Li and his research group in 2005, who utilized aminosulfonic acid under 250 W to carry out the arylation of aldehydes with indoles [136]. Aminosulfonic acid proved to be the best choice for higher product conversion rates between a group of other Lewis and Brønsted acids, so the optimum reaction conditions were studied next. It was discovered that targeted BIMs were synthesized more efficiently, when a mixture of ethanol and water (3:2) was used as the solvent, with the amount of catalyst needed being 150 mol %. Reactions reached completion after 15–360 minutes, depending on the reagents. A wide selection of aromatic and aliphatic aldehydes, substituted indoles and ketones provided encouraging yields of isolated products, ranging from 45% to 95%. Αromatic aldehydes bearing electron-donating substituents underwent the reaction at a much faster rate than other substrates with the exception of nitrobenzaldehydes, which displayed poor solubility thus, emphasizing the generality of this protocol. The drawbacks of a high, non-recyclable catalyst loading and the lack of reactivity of aliphatic ketones under these reaction conditions were issues that could be addressed by other methodologies in the future [136].

**Scheme 32:**
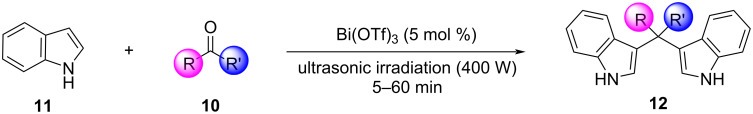
Catalytic approach introduced by Muhammadpoor-Baltork et al.

In 2006, Muhammadpoor-Baltork et al., wanting to eliminate some of the issues of the previous approach, made use of Bi(OTf)_3_ as a relatively less toxic and environmentally friendly catalyst, under ultrasound irradiation (400 W) (Scheme 32) [137]. The amount of catalyst required to reach optimum product yields was significantly lower (5 mol %) with acetonitrile emerging as the most effective reaction medium with ultrasound irradiation having much faster reaction rates, compared to conventional heating techniques, and a wider substrate scope. All aromatic, aliphatic or heterocyclic aldehydes afforded excellent yields of 80–99% in just 5–10 minutes of reaction. Ketones also afforded the corresponding BIMs in good yields, although they required longer reaction times (60–75 min), managing to provide a solution to the drawbacks presented earlier. Nevertheless, the amount and choice of the solvent used (10 mL) and the acidic catalyst had more room for improvement, regarding their environmental impact [137].

**Scheme 33:**
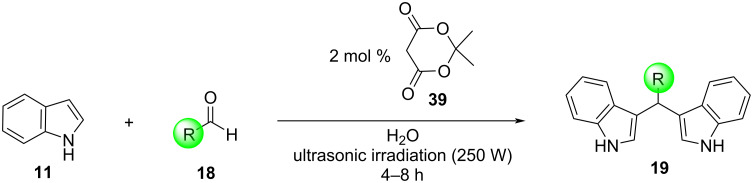
Reaction conditions used by Xiao-Ming.

The next one to incorporate sonochemistry in the Friedel–Crafts bisarylation of aldehydes with indoles in 2008 was Su and his research group, who utilized an ultrasonic bath at a frequency of 40 kHz (250 W) (Scheme 33) [138]. Meldrum’s acid derivative **39** was used as the catalyst (2 mol %) to facilitate the arylation, while water proved to be the best solvent, leading the reaction to competition in 4 to 8 hours, depending on the reagents used. Excellent yields of 63–95% were achieved for both aromatic and aliphatic aldehydes, however, ketones did not show the same reactivity, as even after longer exposure periods under ultrasonic irradiation, only traces of the desired products were detected. Lastly, control experiments revealed that in the absence of either the catalyst or the irradiation the product yields were significantly lower, indicating that they both play a critical role in the catalysis of the reactions. This protocol offered a green solvent (water) for the reaction, while only needing a relatively low amount of catalyst, however, the low reactivity of ketone substrates and the use of an acid for the catalysis of the reaction even at lower yields presented a challenge to be overcome [138].

Li et al. attempted to build upon the employment of aqueous media, leading them to present the use of dodecylbenzenesulfonic acid (ABS) as a proton acid catalyst in water for the formation of BIMs [139]. An amount of 0.5 mol % of ABS was enough to achieve a yield of 97% of *p*-Cl-BIM within 10 minutes, improving both the reaction rates and product yields compared to the reported reaction catalyzed by Meldrum’s acid. Absence of ultrasound irradiation resulted in a significant increase in the reaction times and a slight drop in product yield. This indicated the importance of sonochemistry for the acceleration of the reaction rates, while changes in the irradiation frequency had no observable effect on the reaction. Several aromatic aldehydes were subsequently tested both with and without ultrasound, where the divide in reaction rates was made even more evident for substrates containing electron-donating substituents that required more than 3 hours to form the corresponding BIM. The drawback of the ABS approach is the quite limited substrate scope, since the formation of aliphatic BIMs or the use of ketones as substrates was not achieved, hindering many potential applications in medicinal chemistry [139].

In 2013, Nikpassand et al. replaced the use of an acid with a Schiff base complex, which is a green catalyst capable of promoting the synthesis of BIMs from aldehydes and indoles [140]. 50 mg of the complex were sufficient for the reaction to reach completion after just 5 minutes with ethanol proving to be the optimum solvent. The reaction mixture was irradiated in a water bath at 60 °C by ultrasound (45 kHz or 280 W) with isolated product yields ranging around 85–95%. Except from the shorter reaction times, this method also offered better sustainability, since the Schiff’s base could be recovered from the reaction mixture in a facile way through a simple filtration with ethanol and without losing any catalytic activity in the first five cycles. Notwithstanding the benefits over the previous protocol, the use of ethanol instead of water, the need for heating and the limited substrate scope of just aromatic aldehydes, held back the environmental impact of this proposal [140].

**Scheme 34:**
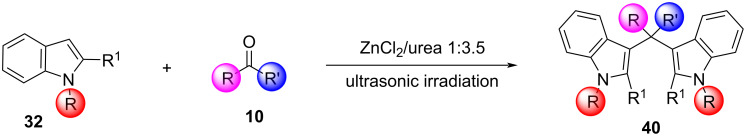
Ultrasonic irradiation-based protocol published by Saeednia.

Two years later, Saeednia and her research team replaced both the catalyst and the solvent of the reaction with a eutectic solvent comprised of ZnCl_2_ and urea in an analogy of 1:3.5, which worked more efficiently than several traditional acids for the synthesis of BIMs (Scheme 34) [141]. The optimum reaction parameters involved the use of 1 equivalent of the eutectic solvent at 60 °C, for a period of 10 minutes with yields of isolated BIMs reaching 92% and being much greater than the respective thermal protocol, where the maximum yield approached 65%. A wide selection of aromatic aldehydes was tested, and it was observed that electron-withdrawing substituents accelerated the reaction rates compared to electron-donating substituents. Ketones and substituted indoles also reacted without a significant change in yield, however, no aliphatic aldehyde substrates managed to form the respective BIMs. Some other benefits that the incorporation of the eutectic solvent provides is the ability of catalyst recycling for up to three cycles, before a noticeable drop in yield is noticed and the ease in product isolation, since a simple filtration with water and a recrystallization from ethanol separates the product from the solvent [141].

**Scheme 35:**
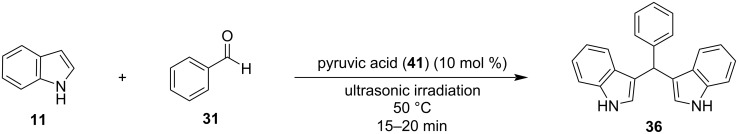
Pyruvic acid-mediated synthesis of BIMs proposed by Thopate.

More recently, in 2022, Thopate and his research group employed pyruvic acid (**41**) as a metal-free biodegradable catalyst, which under ultrasound irradiation in water can prove to be a superior alternative method for the synthesis of BIMs (Scheme 35) [142]. This constituted the first report of the catalytic potential of pyruvic acid, so extensive research to discover the optimum reaction conditions was conducted. 10 mol % of pyruvic acid proved sufficient at 50 °C, while higher catalyst loadings and temperatures did not affect the conversion of the product formed. In the absence of irradiation and at room temperature, reaction rates were significantly lowered, while lack of pyruvic acid completely halted the synthesis of BIMs. With the optimized conditions in hand, various aromatic aldehydes were tested for their reactivity with little to no variation in product yields (85–95%) and reaction times (15–20 min) being observed, depending on the substituents on the benzene ring. Substituted indoles also displayed similar results, while ketones failed to provide substantial product conversions. Thus, an approach utilizing both an environmentally benign catalyst and solvent was developed with the holdback of the need for heating at 50 °C and the inability to synthesize any aliphatic BIMs [142].

#### Ionic liquids

The development of greener, more economical and environmentally benign processes is one of the main challenges of modern chemistry. An environmentally safe alternative that has gained traction in recent years, in lieu of toxic organic solvents, are ionic liquids [143–144]. Ionic liquids enhance reaction rates and product yields, while also being easily recovered and optimized by exchanging the properties of the anions and cations. The properties mentioned render ionic liquids as excellent catalyst choice for greener catalytic processes in organic synthesis [145–146].

**Scheme 36:**
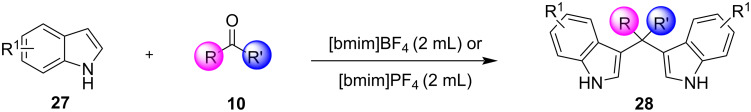
Synthesis of BIMs using [bmim]BF_4_ or [bmim]PF_6_ ionic liquids.

In 2003, Yadav and his research group described a facile and efficient procedure for the preparation of BIMs in 1-butyl-3-methylimidazolium tetrafluoroborate ([bmim]BF_4_) or 1-butyl-3-methylimidazolium hexafluorophosphate ([bmim]PF_6_) ionic liquid (Scheme 36) [147]. The mild reaction conditions of this methodology enabled a variety of substituted aromatic aldehydes to afford their corresponding products in excellent yields. In general, the substituent present on the benzene ring of the aldehyde affects the reaction profile, with electron-withdrawing groups affording superior yields (85–94%) after just 10 minutes. The only exception was the nitro group, which required prolonged reaction times to achieve similar results. Furthermore, the reaction of indole with ketones was also achieved with satisfying yields [147]. The catalytic role of the ionic liquid was made evident, when conducting the reaction in its absence, where no product formation was observed. The benefits offered by the [bmim]BF_4_ and [bmim]PF_6_ ionic liquids are their high recyclability and facile product extraction, which limits the waste produced by this approach [147].

**Scheme 37:**
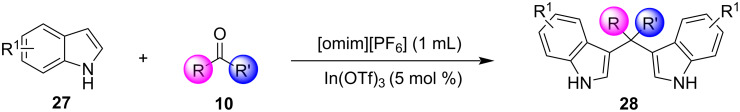
Synthesis of BIMs utilizing In(OTf)_3_ in octylmethylimidazolium hexafluorophosphate as ionic liquid.

The same year, Lohs’ group developed an efficient protocol for the electrophilic substitution reaction of indoles with various aldehydes catalyzed by Lewis acids in octylmethylimidazolium hexafluorophosphate ([omim][PF_6_^–^]) ionic liquid (Scheme 37) [148]. With the optimum reaction conditions in hand, various aldehydes were tested, with both aliphatic and aromatic aldehydes giving the products **28** in high yields (73–96%) [148].

**Scheme 38:**
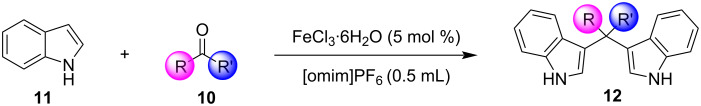
FeCl_3_·6H_2_O-catalyzed synthesis of BIMs with use of ionic liquid.

It is well known that Fe^III^ salts catalyze many organic transformations, including oxidation of sulfides, Michael reactions, thia-Fries rearrangements and synthesis of diphenylmethane and 1,6-anhydroglucopyranoses. In 2004, the same group introduced an alternative protocol for the synthesis of BIMs, utilizing Fe^III^ in an ionic liquid as the catalyst (Scheme 38) [149]. It is worth noting that the FeCl_3_·6H_2_O/[omim]PF_6_ catalytic system can be recovered and reused with a simple extraction, at least four times without significant loss in activity. Subsequently, various aldehydes were studied under optimized conditions. In all cases, both aromatic and aliphatic aldehydes reacted smoothly with indoles in high yields (78–98%). Moreover, this method is highly chemoselective, as ketones do not participate in the reaction, which proved to be useful in the separation of aldehydes from ketones through the synthesis of bis(indolyl)methanes [149].

**Scheme 39:**
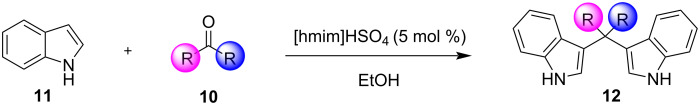
Synthesis of BIMs utilizing the [hmim]HSO_4_/EtOH catalytic system.

In 2005, Loh’s research group also developed another protocol for the synthesis of BIMs, catalyzed by recycled acidic ionic liquid at room temperature (Scheme 39) [150]. Room temperature ionic liquids (RTILs) have many beneficial properties, including high recoverability, advantageous solubility and low toxicity. A catalyst loading of 5 mol % of [hmim]HSO_4_ was sufficient to reach reaction completion after 3.5 hours between benzaldehyde and indole. After this period, the ionic liquid could be recovered and reemployed for up to 5 times, before noticeable decline in catalytic efficiency was observed [150].

**Scheme 40:**
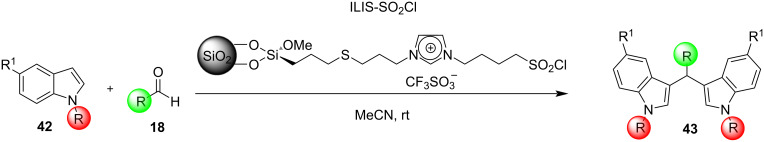
Synthesis of BIMs utilizing acidic ionic liquid immobilized on silica gel (ILIS-SO_2_Cl).

In 2007, Hagiwara et al. developed a new organocatalytic protocol for the reaction of aldehydes with indoles in the presence of acidic ionic liquid immobilized on silica gel (ILIS-SO_2_Cl) (Scheme 40) [151]. This protocol is efficient, mild, practical and recyclable. This approach offers the benefit of a wide substrate scope as both substituted aliphatic and aromatic aldehydes, as well as indoles, form their respective products in excellent yields, even when bearing more acid-sensitive substituents, which can be deprotected in harsher conditions [151].

**Scheme 41:**
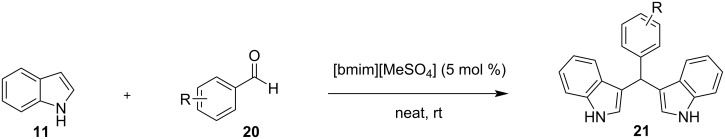
The [bmim][MeSO_4_]-catalyzed reaction of indole with various aldehydes.

In 2008, Chakraborti et al. tested a variety of RTILs as catalysts in the reaction between aldehydes with indole under solvent-free conditions [152]. The ionic liquid [bmim][MeSO_4_] was found to be the most effective catalyst, forming the desired BIMs in excellent yields (85–94%), in very short reaction times (5–30 min) and without the need for conventional heating (Scheme 41).

**Scheme 42:**
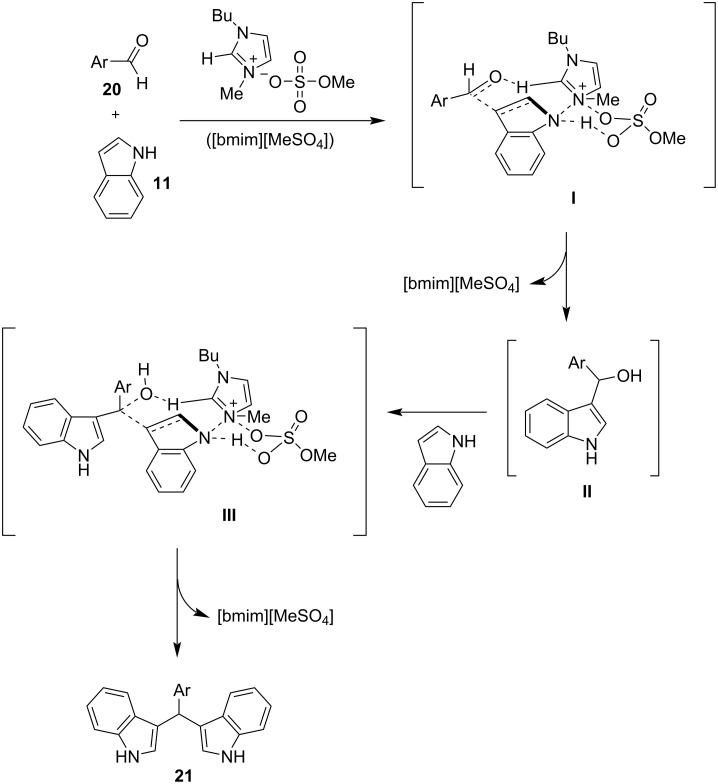
The role of [bmim][MeSO_4_] in catalyzing the reaction of indole with aldehydes.

The mechanism of action of [bmim][MeSO_4_] can be found in Scheme 42. At the beginning of the reaction, the acidic bmim cation forms a hydrogen bond with the carbonyl group, leading to its activation by lowering its LUMO. Meanwhile, the nitrogen atom of the cation interacts with the lone electron pair of the first molecule of indole, leading to a hydrogen bonding between the indole and the MeSO_4_ anion, forming intermediate **I**. Intermediate **I** leads to indolylmethanol **II**, that after activation by [bmim][MeSO_4_] in the same manner as before (with the exception of the hydrogen bonding is formed with the hydroxy group), leading to intermediate **III** that is a precursor of the BIM product **21** [152].

**Scheme 43:**
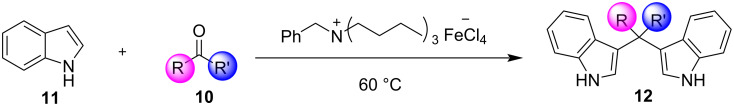
Synthesis of BIMs utilizing FeCl_3_-based ionic liquid ([BTBAC]Cl-FeCl_3_) as catalyst.

In 2009, Veisi et al. reported another method for the synthesis of BIMs utilizing an FeCl_3_-based ionic liquid ([BTBAC]Cl-FeCl_3_) as the catalyst at 60 °C (Scheme 43). The advantages of this method using an FeCl_3_-based ionic liquid are mild reaction conditions, good to high yields, short reaction times (8–30 min), simple work-up procedures, easy preparation of the catalyst and chemoselectivity [153]. Subsequently, in order to challenge the generality of this approach, a variety of aromatic and aliphatic aldehydes were tested, affording the desired products in good to excellent yields ranging from 70% to 98%. It was also noticed that aromatic aldehydes with electron-withdrawing groups formed the desired products faster and in higher yields, compared to electron-donating substituents. The mild reaction conditions also reduced the risk of polymerization of unsaturated substrates, which was an issue in traditional acid-catalyzed methodologies. Ketones required longer reaction times, due to the electron-donating and steric hindrance caused by the methyl group [153].

**Scheme 44:**
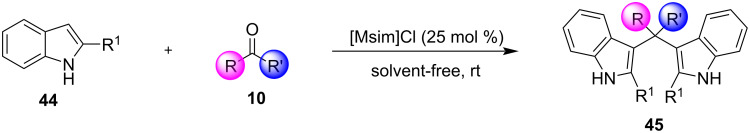
Synthesis of BIMs using [Msim]Cl at room temperature.

In 2010, Zolfigol et al. introduced a protocol based on a Brønsted acid ionic liquid, 3-methyl-1-sulfonic acid imidazolium chloride ([Msim]Cl), which can act as an easily synthesized and relatively cheap catalyst for the Friedel–Crafts reaction between aldehydes and indoles under solvent-free conditions (Scheme 44) [154]. The substrate scope of this approach was astonishing as all types of substituted aldehydes, ketones and indoles formed the respective BIM products in excellent yields of 76–96% in just 10–90 seconds without any difficulty. While the impressive reaction rates and wide substrate scope in solvent-free conditions elevate this protocol, the relatively high catalyst loading of the IL can prove detrimental [154].

**Scheme 45:**
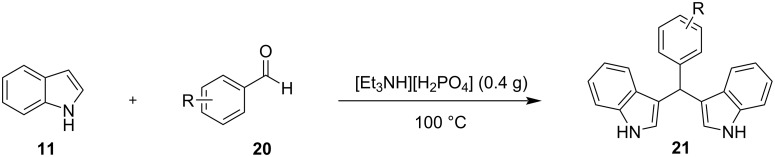
[Et_3_NH][H_2_PO_4_]-catalyzed synthesis of bis(indolyl)methanes.

In 2012, another cheap and mild acidic ionic liquid was used by Kalantari as a catalyst for the reaction between aldehydes and indoles (Scheme 45) [155]. Triethylammonium dihydrogen phosphate-[Et_3_NH][H_2_PO_4_] is a mild Brønsted-acidic ionic liquid that was found to be an efficient catalyst for this reaction. After optimizing the conditions, the generality of this approach was examined, employing several aromatic aldehydes. The results showed that the reaction proceeds very efficiently in all cases with yields ranging from 84–98% after just 5–30 minutes with the drawback of the necessity of conventional heating [155].

**Scheme 46:**
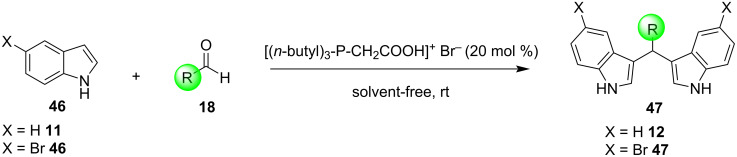
PILs-catalyzed synthesis of bis(indolyl)methanes.

In 2013, Khazaei et al. successfully introduced a green phosphonium ionic liquid (PIL) as an alternative to toxic solvents [156]. Tributyl(carboxymethyl)phosphonium bromide can be utilized as an efficient and green catalyst for the synthesis of BIMs without the need of an organic solvent or heating, greatly diminishing the energy consumption of this approach (Scheme 46). Various substituted indoles and aldehydes managed to from the respective BIMs in satisfying yields (75–96%) in just 15 to 120 minutes, however, the employment of ketones was not mentioned. As in most ionic liquid-based methodologies, the work-up procedures are simple and toxic byproducts are scarce. However, the cost of the catalyst preparation can hold back more widespread applications [156].

**Scheme 47:**
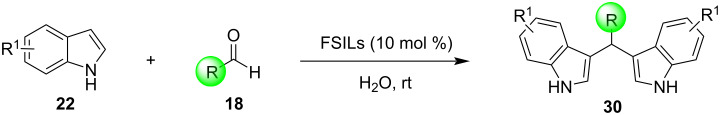
FSILs-mediated synthesis of bis(indolyl)methanes.

In 2014, Zhang and co-workers developed a new protocol for the synthesis of BIMs, employing new polyacrylonitrile fiber supported ionic liquids (FSILs) at room temperature, in water (Scheme 47) [157]. Most substituted aldehydes and indoles successfully took part in the reaction affording the isolated products in yields ranging from 90% to 96%, in 3 to 8 hours with aliphatic substrates displaying the lowest reaction rates. The catalysts were easily recovered and reemployed for up to 10 times, before a noticeable drop in product yield was observed, which in tandem with the use of water as the solvent aided of the environmental impact of this methodology. However, the lack of reactivity of ketones and larger aliphatic aldehydes were some drawbacks observed in this approach that could be later improved upon [157].

**Scheme 48:**
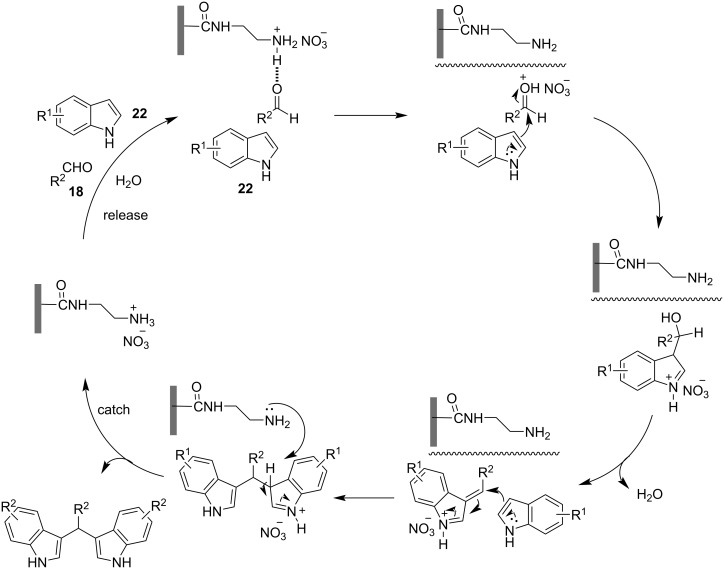
Possible “release and catch” catalytic process.

The mechanism of action proposed by Zhang is presented in Scheme 48. As the reaction begins, FSIL (in this scheme ethylammonium nitrate FSIL) activates the carbonyl group through the formation of a hydrogen bond, which facilitates the nucleophilic attack of the first indole. Subsequently, while the intermediate formed after the addition is stabilized by the disassociated nitrate, another indole attacks the intermediate to form the desired BIM. The FSIL is regenerated after the ethylamine recovers the proton and the nitrate group regenerates the catalyst, which can now be reused for another catalytic cycle [157].

**Scheme 49:**
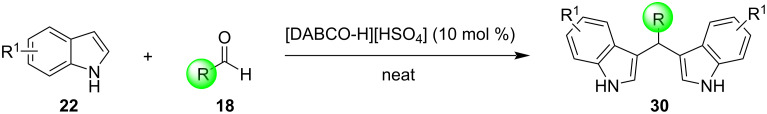
Synthesis of bis(indolyl)methanes by [DABCO-H][HSO_4_].

Two years later, Xu and co-workers proposed the employment of a 1,4-diazabicyclo[2.2.2]octane (DABCO)-based ionic liquid as the catalyst in the synthesis of BIMs (Scheme 49) [158]. The Friedel–Crafts arylation was performed under optimized solvent-free conditions with a catalyst loading of 10 mol %, proving sufficient in order to obtain the desired products in high yields (61–98%) in 0.7–2 hours. After the reaction’s completion, the catalyst could be retrieved in a facile manner and reemployed for six catalytic cycles, before a loss in product yield was noticeable, which combined with the added benefit of the lack of conventional heat, elevated this approach [158].

**Scheme 50:**
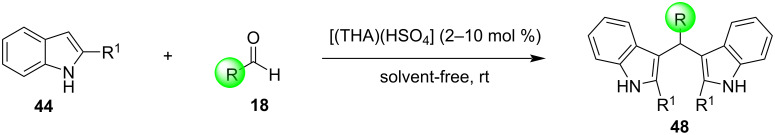
Synthesis of bis(indolyl)methanes by [(THA)(SO_4_)].

In 2017, Honarmand et al. utilized tris(hydroxymethyl)methaneammonium hydrogensulfate [(THA)(HSO_4_)] as the first nanoaliphatic ammonium-based ionic liquid as a catalyst for the synthesis of BIMs at room temperature and under solvent-free conditions (Scheme 50) [159]. This ionic liquid is green, environmentally friendly and recyclable, and the advantages of this method are high yields (84–99%), cleaner reaction profile, fast reaction rates (3–35 min), the avoidance of organic solvents and facile work-up [159].

**Scheme 51:**
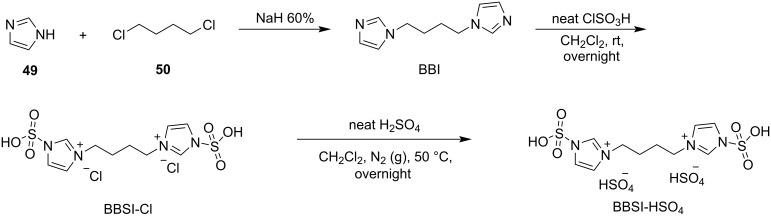
Synthesis of BBSI-Cl and BBSI-HSO_4_.

**Scheme 52:**
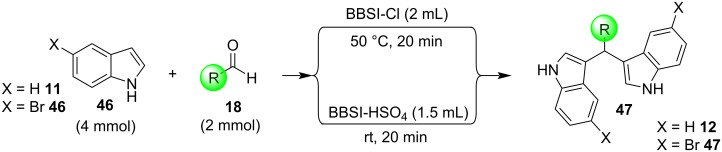
Synthesis of BIMs in the presence of BBSI-Cl and BBSI-HSO_4_.

**Scheme 53:**
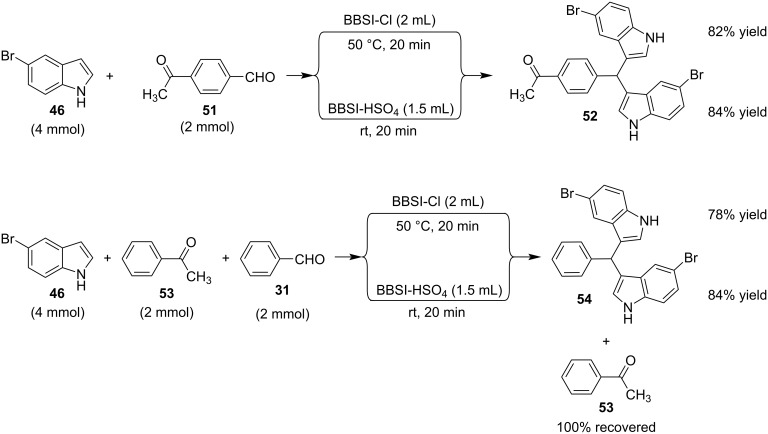
Chemoselectivity of the present method.

In 2018, Khaligh and co-workers synthesized two new binuclear sulfonic-functionalized ionic liquids (TSIL) [1,1′-butylenebis(3-sulfo-3*H*-imidazol-1-ium) chloride – BBSI-Cl and 1,1′-butylenebis(3-sulfo-3*H*-imidazol-1-ium) hydrogen sulfate – BBSI-HSO_4_] with chloride and hydrogen sulfate as the counter anions (Scheme 51) [160]. The physical properties of the new TSILs were determined and their dual solvent–catalytic activity was investigated for the synthesis of the symmetrical BIMs under mild conditions (Scheme 52) [160]. The current protocol has benefits, such as simple and sustainable experimental procedures, good isolated yields of the desired products, short reaction times, satisfactory recyclability and chemoselectivity of the employed ionic liquids (Scheme 53) [160].

**Scheme 54:**
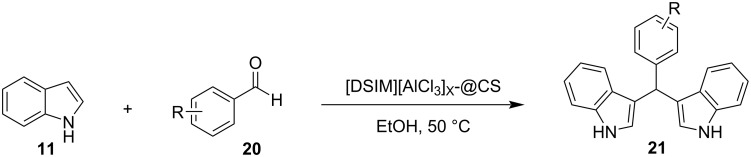
Synthesis of BIMs catalyzed by chitosan-supported ionic liquid.

Among the solid support materials, chitosan and chitosan natural biopolymer, are excellent support materials because of their exclusive chelating and mechanical properties such as unique three-dimensional structure, biodegradable nature and chemical reactivity. Konda and co-workers have developed an alternative methodology for the synthesis of BIMs, utilizing chitosan-supported ionic liquid (CSIL) [161]. After optimizing the conditions, the generality of this protocol was examined, employing several aromatic aldehydes in high yields (80–95%). The chitosan catalyst as well as the ionic liquid solvent could be recovered in a facile manner after being used in the reaction and could be reemployed several times without significant degradation in catalytic efficiency (Scheme 54).

**Scheme 55:**
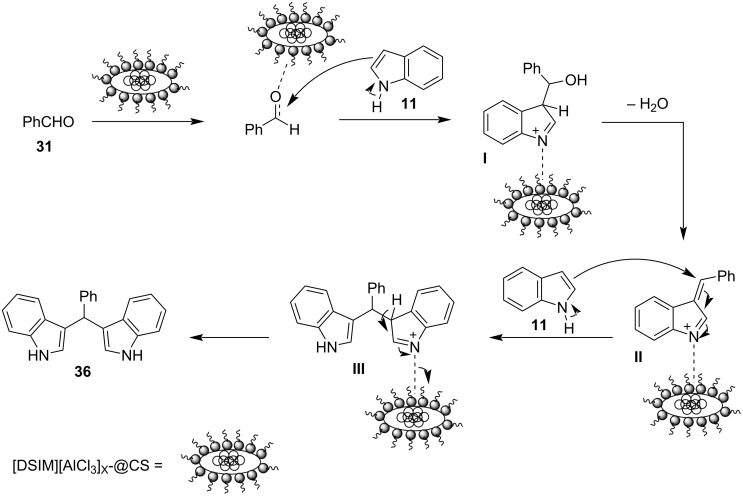
Proposed mechanism of action of CSIL.

The reaction mechanism proposed by Konda and his co-workers can be seen in Scheme 55. As in most previous examples, the first step of the catalytic cycle involves the activation of the carbonyl group by the catalyst’s surface, which allows the nucleophilic attack by the first indole to occur forming adduct **I**. Following a dehydrogenation, which leads to the intermediate **II**, another molecule of indole attacks the CSIL-stabilized **II** to form intermediate **III**, which in turn leads to the desired BIM product **36** after a simple rearrangement.

#### Eutectic solvents

Solvents play a crucial role in green chemistry, because they are often the largest source of pollution in chemical transformations. Nowadays, researchers are shifting their focus towards limiting the employment of organic solvents and discovering greener alternatives to combat this phenomenon. As mentioned above, one of the alternatives proposed were room temperature ionic liquids, which immediately attained a privileged place in organic synthesis [162–164]. In the last decade, another alternative reaction medium has gained attraction, known as deep eutectic solvents (DESs). Abbott et al. were the first to dabble in the research of these solvents, which are formed by mixing solid organic salts with hydrogen-bond-donating organic complexes (such as urea derivatives) [165–166]. Some of the advantages offered by DESs include low toxicity, ease of preparation and biodegradability, while having the ability to act both as solvents and catalysts for several organic reactions [167–170].

**Scheme 56:**
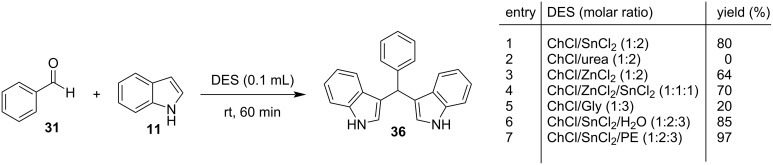
Optimization of the reaction in DESs.

**Scheme 57:**
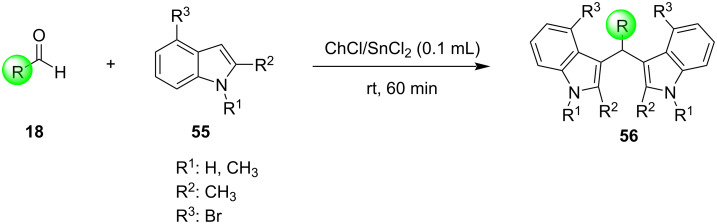
Synthesis of BIMs using ChCl/SnCl_2_ as DES.

In 2012, Azizi et al. developed a general protocol for the synthesis of bis(indolyl)methanes in deep eutectic solvents [171]. First, they tested different reaction conditions in the reaction between indole **11** and benzaldehyde **31** in five deep eutectic solvents (Scheme 56). It was observed that the best yield (90%) of isolated product was achieved when employing 0.1 mL of the choline chloride/SnCl_2_ eutectic solvent in polyethylene glycol after 20 min without utilizing conventional heating or other additives (Scheme 56). With the optimized reaction conditions in hand, a variety of aromatic aldehydes, bearing electron-withdrawing and electron-donating groups, and substituted indoles were screened, affording the products in good to excellent yields (64–97%) (Scheme 57).

**Scheme 58:**
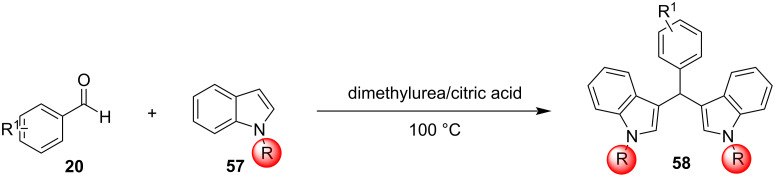
Synthesis of BIMs derivatives in presence of DES.

In 2014, Khabazzadeh and co-workers presented a dimethylurea/citric acid deep eutectic solvent to act as the catalyst in the synthesis of BIMs [172]. This DES provided the optimum results, when it was synthesized by mixing dimethylurea and citric acid in a 6:4 ratio at 100 °C, and then adding the reagents in the reaction mixture. Having in hand the optimum reaction conditions, a variety of carbonyl compounds with indole **57** were tested, affording the desired products **58** in excellent yields (86–96%) (Scheme 58). This method offered several advantages, including the employment of a green DES instead of organic solvents, high yields, short reaction times, a simple work-up procedure and reusability of DES.

**Scheme 59:**
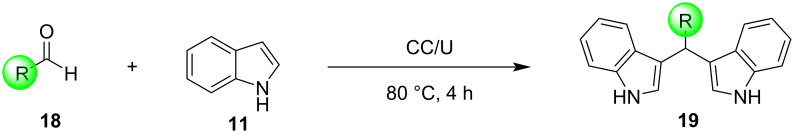
BIMs synthesis in choline chloride/urea (CC/U).

The same year, Handy et al. have demonstrated that choline chloride/urea (CC/U) is a useful solvent for the synthesis of bis(indolyl)methanes under relatively mild conditions (Scheme 59) [173]. Wanting to discover the optimum reaction conditions, the authors first studied the reaction between *p*-anisaldehyde and indole, where a yield of 96% was achieved after 4 hours at 80 °C with the CC/U system acting as the reaction medium. Various substituted aromatic and aliphatic aldehydes were tested in these conditions with great efficiency and yields ranging from 80% to 99% for both electron-donating and electron-withdrawing substituents. Moreover, the recyclability of the eutectic solvent was also challenged and it was discovered that the CC/U system could be reused for up to 5 catalytic cycles, before a drop in efficiency was observed [173].

#### Flow chemistry

Flow chemistry has received remarkable attention in recent years as an innovative technology that can be utilized in organic catalysis. Continuous flow catalysis uses a continuous stream of various reagents, introduced by pumps into the reaction mixture, while it is mixed by a flow reactor. Compared to other methods of catalysis, flow chemistry offers a much greater control over reaction parameters, which can be modified easily, while reducing chemical waste and allowing more precise reproducibility. With all these benefits, it did not take long for the implementation of flow chemistry in the synthesis of BIMs as an alternative, environmentally benign, catalytic protocol [174–175].

**Scheme 60:**
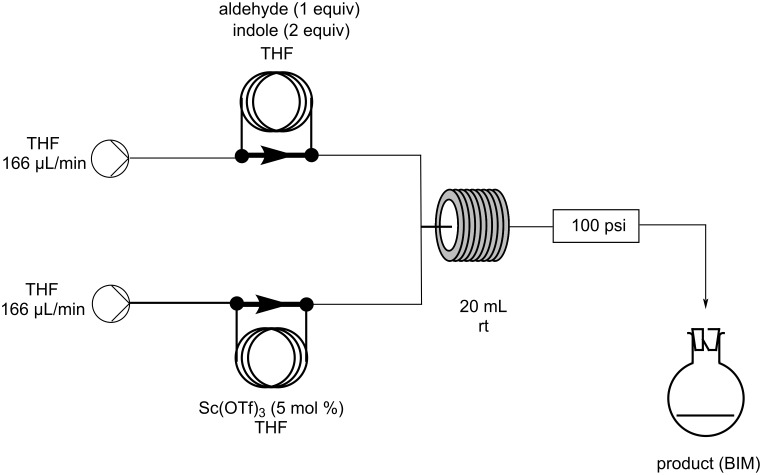
Flow chemistry-based synthesis of BIMs by Ley.

The first application of flow chemistry for the Friedel–Crafts reaction between aldehydes and indoles was introduced by Ley and his research group, in 2017, who picked Sc(OTf)_3_ as the acidic catalyst (Scheme 60) [176]. The first step of their research involved the optimization of the reaction conditions between benzaldehyde and indole. It was discovered that when tetrahydrofuran (THF) was used as the solvent in a 5 mL reaction coil at room temperature with a catalyst loading of 5 mol % and with a reaction time of 45 minutes, the largest yield (92%) of the isolated BIM was achieved, while increases or decreases in flow rates reduced or did not affect the product yield any further. The reaction proceeded smoothly for most aromatic aldehydes and substituted indoles, apart from pyridinecarboxaldehyde derivatives, reaching yields of isolated BIMs of 65–96%. An attempt at a larger scale reaction (29 mmol) was also successful, proving the generality of this protocol. However, the drawbacks of this methodology included the high investment cost of the continuous flow equipment at the time and the lack of reactivity of ketones and aliphatic aldehydes [176].

**Scheme 61:**
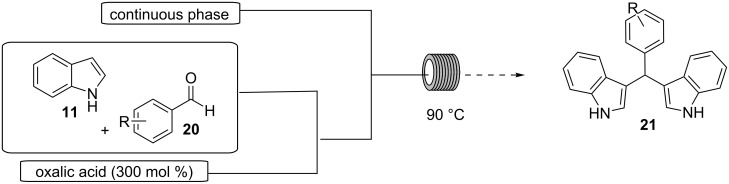
Flow chemistry-based synthesis of BIMs proposed by Nam et al.

Three years later, Nam et al. attempted to utilize oxalic acid for the role of the catalyst, since it can be dissolved in water which would render their approach more sustainable (Scheme 61) [177]. Promising yields were attained with the use of water as the solvent, however, difficulties were encountered, since the reagents were not soluble in water, causing clogging thus reducing the stability of the process and damaging the equipment used. To combat this issue, the solvent was switched to a mixture of DMF and water, where both starting materials exhibited satisfying solubility and the single-phase continuous flow model was dropped for the droplet-based model, which expels both the solids and the continuous phase simultaneously, eliminating the risk of clogging. After overcoming this issue, the focus turned to the optimization of the reaction conditions. Different mineral oils were tested for the continuous phase with perfluoro-2-*n*-butyltetrahydrofuran), emerging as the best option, providing the best yields and highest reaction rates of only 20 minutes to reaction completion, while also being easily recoverable with a single filtration. The reaction mixture could also be heated at 95 °C and 3 equivalents of oxalic acid could be added to reach 87% of isolated yield for the reaction between indole and benzaldehyde. Many substituted aromatic aldehydes and indoles were tested, all providing satisfying yields, ranging from 68% to 87% with good process stability and with the option to also form the respective tris(indolyl)methanes (TIMs), when adjusting the analogy between DMF and water of the solvent. In this manner, the use of a greener catalyst was achieved with the drawback of a higher catalyst loading, compared to the previously reported approach [177].

#### Aminocatalysis

**Scheme 62:**
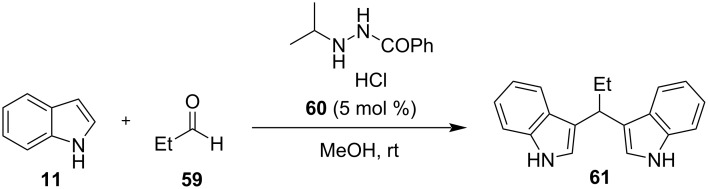
Amino-catalyzed reaction of indole with propionaldehyde.

With the advent of green chemistry and the increased environmental awareness of organic chemists, aminocatalysis has become an extremely appealing catalytic methodology for numerous organic transformations [178–181]. Aminocatalysis was always a subject of interest in the scientific community, due to the uniqueness of the interactions between the catalyst’s architecture and activity, with iminium ion catalysis, especially, being in the spotlight [182–183]. The first aminocatalytic protocol for the synthesis of BIMs was introduced in 2005 by Gibbs et al. [184], who employed benzoic hydrazide derivative **60** as the catalyst, due to its previously reported applications in various aminocatalytic applications (Scheme 62) [184]. Methanol was discovered to be the best choice of solvent, as the reaction would not proceed favorably in non-polar solvents, with 5 mol % of the benzoic hydrazide derivative **60** proving sufficient to reach product yields of 84% after 24 h. The role of catalyst was also proven as the reaction did not occur in the absence of the hydrazine. The substrate scope was also impressive as both substituted aldehydes, ketones and indoles successfully formed their respective BIMs at respectable yields (50–84%) in similar reaction times. While the yields and the reaction rates had room for improvement, this protocol showcased the benefits of aminocatalysis in the synthesis of BIMs and other molecules with interesting pharmacological activities [184].

**Scheme 63:**
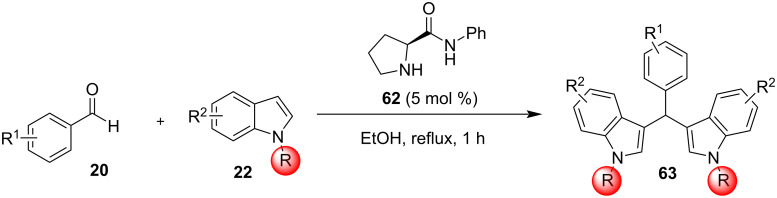
Aminocatalytic synthesis of BIMs.

In 2020, Basumatary et al. incorporated ʟ-prolineamide **62** for the amino-catalyzed synthesis of BIMs, in place of ʟ-proline, which displayed issues of high catalyst loading and lower reactivity (Scheme 63) [185]. An amount of 5 mol % of ʟ-prolineamide **62** was employed under reflux conditions (80 °C) with ethanol acting as the optimum solvent for the model reaction between benzaldehyde and indole, where a yield of 93% was attained after 1 hour. Several aromatic and aliphatic aldehydes **20** reacted successfully in the presence of the aminocatalyst with excellent isolated product yields ranging from 84% to 93% without any significant fluctuations being observed between the various reagents even when substituted indoles were utilized.

**Scheme 64:**
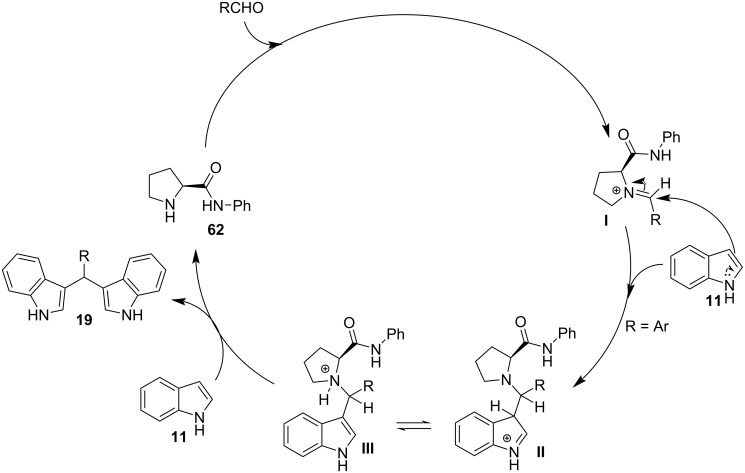
Proposed mechanism for the aminocatalytic synthesis of BIMs.

After mechanistic studies, a catalytic pathway was proposed beginning with the reaction of the catalyst with the aldehyde forming the iminium salt **I** (Scheme 64). Subsequently, a nucleophilic attack by a first indole occurs leading to intermediate **II**, where after a rearrangement and another nucleophilic attack by a second indole, product **19** is finally obtained.

#### Biocatalysis

Recently, lipases have been involved in an increasing number of C–C bond-formation reactions, due to their high adaptability, low toxicity, and ability to be employed in both organic and aqueous environments without drops in efficiency [186–190]. Le and co-workers [191] were the first to make use of biocatalysis in the synthesis of BIMs, utilizing α-chymotrypsin in a mixture of water/ethanol. However, while they managed to highlight the boundless possibilities of biocatalytic protocols, their approach lacked applicability, as only aliphatic and aromatic aldehydes bearing electron-donating substituents could react in moderate yields.

**Scheme 65:**
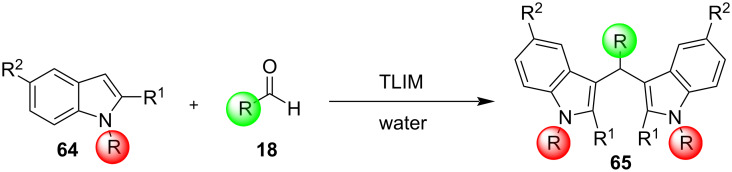
Enzymatic reaction of indole with aldehydes.

**Scheme 66:**
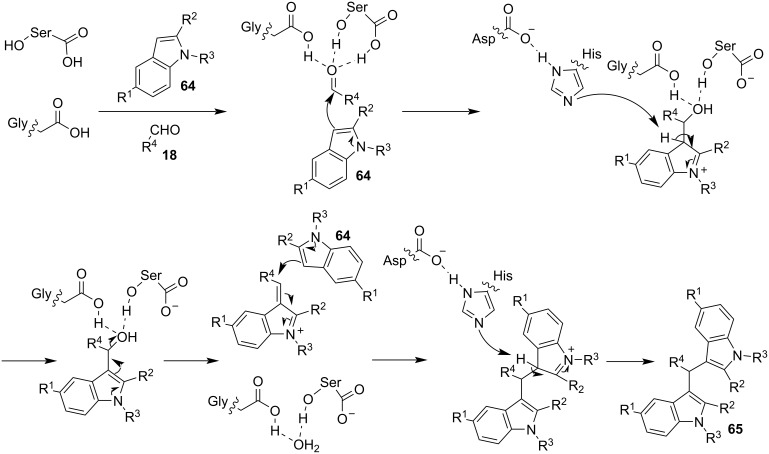
Proposed mechanism for the synthesis of BIMs catalyzed by TLIM.

In 2020, Hu and his research group introduced the use of the lipase TLIM in an aqueous medium, for the catalysis of the Friedel–Crafts arylation of aldehydes to indoles (Scheme 65) [192]. This method is environmentally friendly and highly efficient for the synthesis of these compounds. A wide substrate scope of various aromatic and aliphatic aldehydes, as well as indoles, was employed to great effect and in excellent yields (73–99%). A mechanism of action for the lipase TLIM was proposed by Hu as seen in Scheme 66. At the beginning of the catalytic cycle, the Gly and Ser residues of the active site of the lipase interact with the carbonyl group to form an oxyanion hole, which lowers the LUMO of the electrophilic aldehyde and allows the nucleophilic attack by the first indole molecule. The intermediate product formed is activated by the His residues of the lipase, allowing for a second nucleophilic attack by another indole molecule, which results in the final BIM **65** after a simple rearrangement while the catalyst returns in its original state, ready to participate in a new catalytic cycle [192].

#### Electrochemistry

Electrochemistry, which entails the addition or removal of electrons from molecules through a source of electrostatic potential, has been popularized as a greener approach to organic catalysis, since its inception in the 1830s by Faraday. Electroorganic synthesis is considered an environmentally benign alternative to traditional reagent-based methodologies, due to its wide range of applications, while avoiding the use of toxic catalysts or redox reagents [193].

**Scheme 67:**
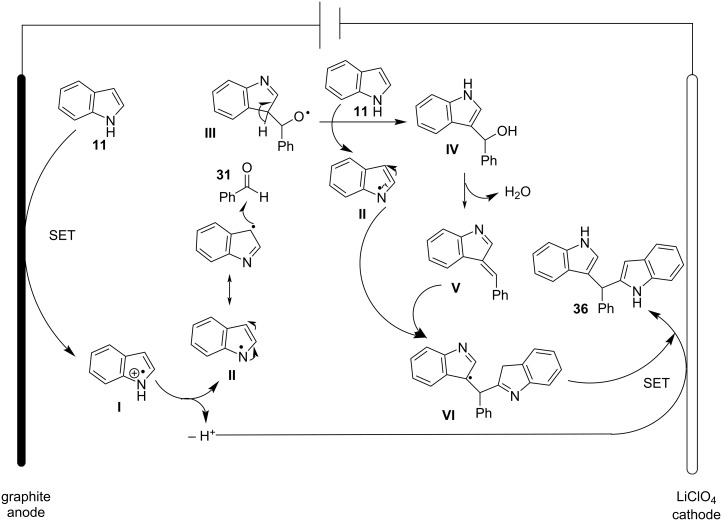
Proposed reaction mechanism by Badsara.

In 2022, Badsara and his research group published a protocol for the synthesis of BIMs that made use of electrochemistry to realize the Friedel–Crafts reaction of aldehydes with indoles [194]. Utilizing a graphite anode and a graphite cathode with LiClO_4_ in acetonitrile at a 10 mA current flow, after 90 minutes, provided the optimum product yield, which ranged from 52% for aliphatic aldehydes to 92% for aromatic substrates. After the generality of the method was confirmed with the employment of varied substituted aromatic and aliphatic aldehydes, control experiments were conducted so a mechanism of action could be proposed. It was discovered that when the reaction was conducted either without the use of a current flow, or in the presence of TEMPO no product was formed, which indicated a radical pathway for the bisarylation and proved that electricity is mandatory for the reaction to proceed. With this information and after more cyclic voltammetry experiments, a possible reaction mechanism was proposed, where the indole is oxidized at the anodic position affording radical cation **I** (Scheme 67), which after release of a proton forms radical **II** (Scheme 67). This radical in turn reacts with the aldehyde and another molecule of indole to form radical **III** (Scheme 67), that after the removal of water reacts with radical **II** (Scheme 67) and subsequently undergoes a cathodic reduction to form the desired BIM **36**. In summary, this protocol provided a catalyst-free approach for the synthesis of BIMs, with high tolerance for functional groups and proceeds at room temperature [194].

#### Photochemistry

Photocatalysis is the branch of organic catalysis which specializes in the acceleration of organic reactions utilizing light as the energy source. Since the introduction of its concept, photocatalysis has become a staple in organic chemistry and is gaining more traction yearly as a green and environmentally benign method for the facilitation of a wide range of organic transformations, due to its renewability, sustainability and low cost [195–199].

**Scheme 68:**
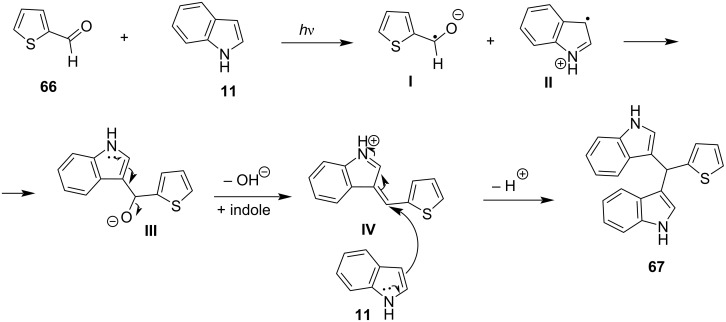
Mechanism proposed by D’Auria.

The first application of photocatalysis for the Friedel–Crafts addition of indoles to aldehydes was developed by D’Auria in 1991, who utilized a high-pressure mercury lamp as the irradiation source [200]. The desired product was formed after 6 hours in acetonitrile at a yield of 30–50%, depending on the substrate. The proposed mechanism of action involved a single-electron transfer (SET), where the formed radicals **I** and **II** (Scheme 68) reacted affording a 3-indolyl carbinol intermediate, which acts as an electrophile towards another molecule of indole for the formation of the final product (Scheme 68) [200].

**Scheme 69:**
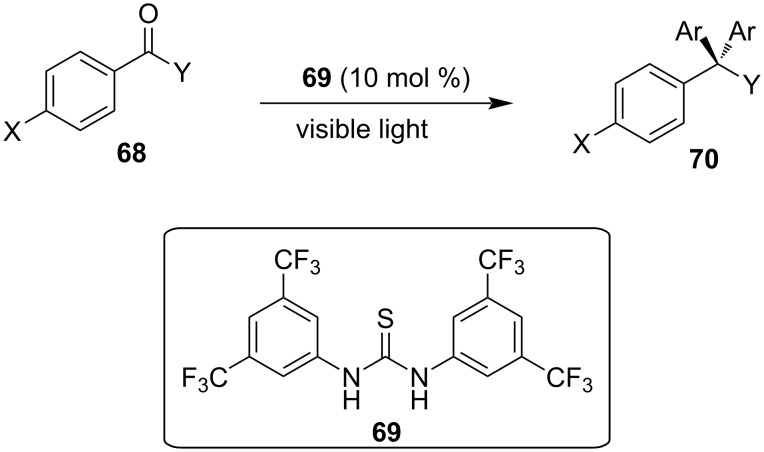
Photoinduced thiourea catalysis.

**Scheme 70:**
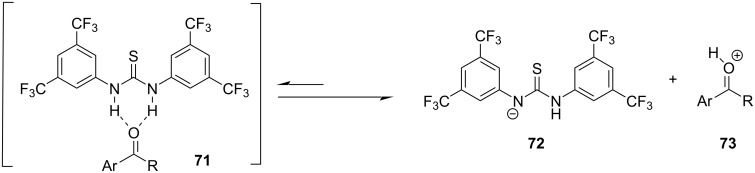
Proposed mechanism of photoacid activation.

In 2019, Badillo and his research group introduced the use of Schreiner’s thiourea **69**, which acts as a photoacid under visible light irradiation (Scheme 69) [201]. Photoacids are molecules that release an acid moiety, in a reversible manner, when irradiated under UV–vis light, presenting an interesting alternative to the use of traditional Brønsted acids [202]. The optimum reaction conditions involved dioxane as the solvent, 10 mol % catalyst loading and 370 nm LED lamps as the irradiation source, while in the dark or without the use of thiourea, no product was observed. Product yields were significantly improved reaching 90%, while substrates that were sterically hindered or had electron-donor substituents displayed slightly lower yields. After several mechanistic experiments, it was proposed that thiourea and the carbonyl group of the aldehyde acted as photoinitiators for the Friedel–Crafts reaction, since thiourea acted as a photoacid and facilitated the double indole addition (Scheme 70) [201].

**Scheme 71:**
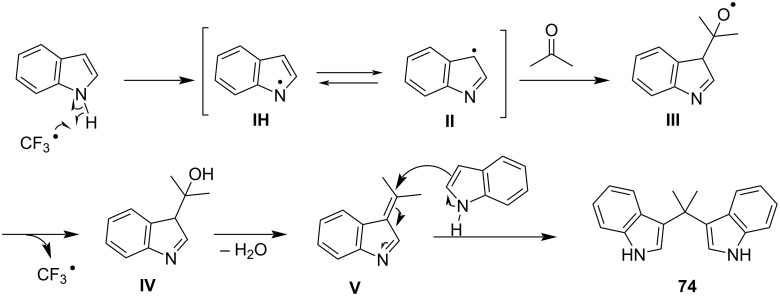
Proposed mechanism of action for CF_3_SO_2_Na.

One year later, in 2020, Qiu and his research group proposed the commercially available CF_3_SO_2_Na as a mediator for the Friedel–Crafts addition giving an alternative to photocatalysts [203]. The maximum yield was achieved after 24 hours, when toluene was used as the solvent, 350–380 nm lamps were the irradiation source and 2 equivalents (200 mol %) of CF_3_SO_2_Na were added. Aldehydes showed high efficiency, yielding products in 76–89%, while ketones highlighted decreased reactivity, leading to yields ranging from 30% for cyclic substrates to 81% for acetone. After the appropriate control experiments, where the necessity of the catalyst and UV irradiation, as well as the N–H bond of indole were confirmed, a mechanism of action was proposed. Following the irradiation of the reaction mixture, CF_3_**^•^** is generated after the catalyst is oxidized by the oxygen of the atmosphere. The CF_3_**^•^** radical subsequently reacts with the amino hydrogen of the indole group affording radical **I** (Scheme 71), which in turn reacts with the carbonyl group forming intermediate **III** (Scheme 71), that after a β-elimination and reaction with a second indole produces the desired BIM. The uniqueness of this catalytic method lies in the activation of the indole group, instead of the carbonyl group, as well as the high tolerance for both ketones and aldehydes [203].

**Scheme 72:**
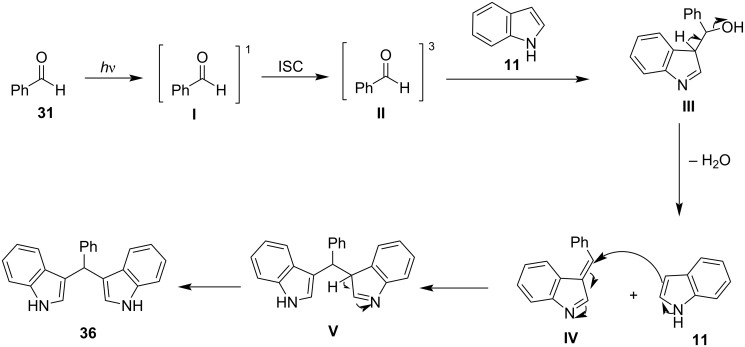
Proposed mechanism for the synthesis of BIMs by Mandawad.

In 2022, Mandawad and his research team introduced a new protocol using a 150 W tungsten lamp for the irradiation of the reaction mixture [204]. This method does not involve the use of a catalyst or solvent, greatly reducing chemical waste and its reaction time is low (30–45 min), giving an alternative greener approach for the synthesis of BIMs. The isolated yield of the products ranged from 80–88%, however, the drawback of this protocol is that the substrate scope only includes aromatic aldehydes, which showcased limited applications. The proposed mechanism of action is similar to the other already published protocols with the exception of the initiation, which takes place with the intersystem crossing of the singlet aldehyde to the triplet state **II** (Scheme 72) [204].

**Scheme 73:**
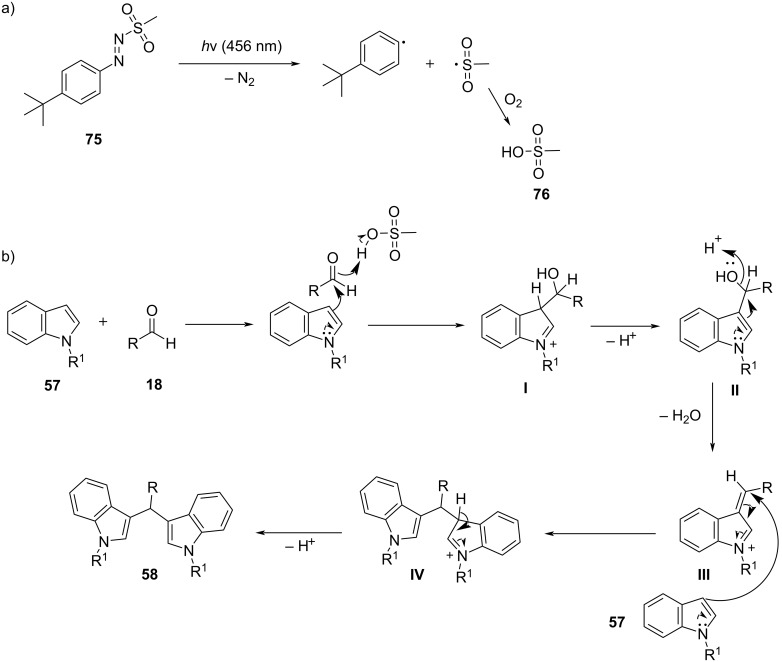
Proposed mechanism for the (a) acid generation and (b) synthesis of BIMs.

The most recent application of photocatalysis was proposed by Kokotos and his research group in collaboration with the PhotoGreen lab at the University of Pavia, and it involves the use of arylazosulfones as photoacid generators (PAGs), which in contrast to photoacids, irreversibly release an acid moiety when irradiated [205]. This protocol utilized 4-*tert*-butyl-azosulfone as the PAG in low catalyst loading (0.5 mol %) for 6–18 hours under blue LED light irradiation (456 nm) with chloroform as the optimum solvent (Scheme 73). A wide variety of aliphatic and aromatic aldehydes and ketones were tested, as well as substituted indoles and the reaction proceeded successfully yielding 38–96% of product, with ketones requiring the longest reaction times. The mechanism of action is based on the formation of methanesulfonic acid, which is derived from the radical CH_3_SO_2_**^•^**, when the arylazo sulfone is excited. After this step, the mechanism follows the same path as an acid-catalyzed Friedel–Crafts addition (Scheme 73). The wide variety of substrates and low catalytic loading is the benefit of this protocol, however, ketones required prolonged reaction times and provided lower yields, due to their decreased reactivity [205].

#### Miscellaneous

**Scheme 74:**
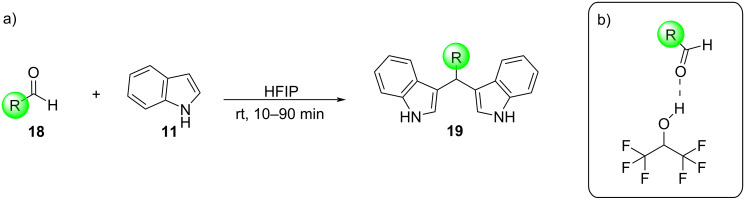
a) Reaction conditions employed by Khaksar and b) activation of the carbonyl group by HFIP.

In 2012, Khaksar and co-workers introduced an efficient and simple synthetic protocol which employed 1,1,1,3,3,3-hexafluoro-2-propanol (HFIP) as both the catalyst and solvent of the reaction (Scheme 74) [206]. HFIP has served in various organic transformations as a hydrogen-bond donor, which rendered it as a strong candidate for the synthesis of BIMs. A model reaction was conducted between indole and benzaldehyde, where after 60 minutes at room temperature, the desired product was formed in a yield of 90%, confirming the ability of HFIP to activate the carbonyl group (Scheme 74). After this confirmation, the universality of this approach was also challenged, where several aliphatic and aromatic aldehydes reacted forming the corresponding BIMs, with equal effectiveness, in yields ranging from 80% to 95%. HFIP could also be separated and recovered by distillation, without a drop in catalytic efficacy, reducing the chemical waste produced considerably. Therefore, this method avoided the use of acids, metals and conventional heating with the drawback of the high amount of the relatively expensive HFIP required as it also plays the role of the solvent [206].

**Scheme 75:**
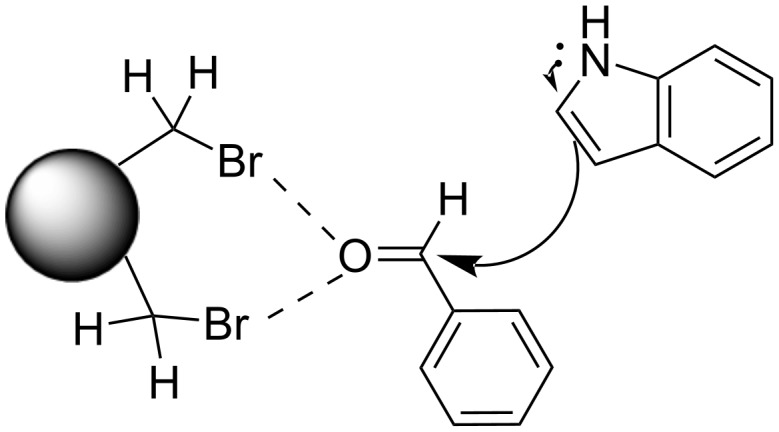
Activation of the carbonyl group by the PPy@CH_2_Br through the formation of a halogen bond.

In 2018, Kim and his research group produced various porous organic polymers in order to study their catalytic activity in the development of organic molecules with interesting pharmacological applications, such as BIMs [207]. At the end, hypercrosslinked polyaromatic spheres (HCPs) proved superior, since they contain unreacted halogen groups in their surface that can catalyze the electrophilic substitution of indoles and aldehydes through halogen bonding (Scheme 75). HCPs are synthesized in a facile manner and also have excellent thermal stability and recyclability, which further enhance their utility. The choice of HCP was polystyrene (PPy)-crosslinked with bromoethyl (CH_2_Br) groups with the interconnected –CH_2_– bridges forming the microporous surfaces. The optimum yields of BIMs were observed under neat conditions at 80 °C with an approximate PPy@CH_2_Br amount of 10 mg added. Diverse aromatic and heterocyclic aldehydes reacted with various substituted indoles with good yields (84–96%) after 1 hour with ketones, however, not sharing the same results. The PPy@CH_2_Br spheres were recovered through centrifugation, where they could be reemployed for up to five runs before drops in product conversion rates were noticed [207].

**Scheme 76:**
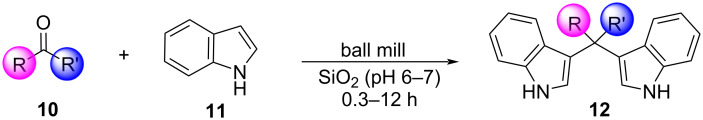
Reaction conditions utilized by Mhaldar et al.

In 2019, Mhaldar et al. published a mechanochemical protocol, which avoided both the use of a solvent and catalyst, significantly decreasing toxic chemical waste production (Scheme 76) [208]. In this methodology, the reactions performed with the ball-milling technique in the presence of SiO_2_, which played the role of both the grinding medium and the acid catalyst, due to its slightly acidic character (pH 6–7). After 20–150 minutes of grinding for arylaldehydes and 420–720 minutes for alkylaldehydes, high yields of product were isolated (62–95%) with analogous reactivity being exhibited by certain ketones. However, inactivated ketones did not react under these conditions and the scalability of this approach was challenging, due to the expense of the ball-mill apparatus [208].

In 2020, Liang et al. studied the potential application of inorganic polymer flocculation materials in the synthesis of BIMs [209]. More specifically polyaluminum chloride (PAC) could be employed in solid-phase grinding conditions, avoiding the contamination problems caused by the use of metal ions or surfactants. 10 mg of PAC were added in tandem with 0.2 g of SiO_2_, which has a dispersing effect and a role as a promoter, enhancing product yields. Substituted aromatic aldehydes reacted successfully with higher conversion rates (80–99%) after 30 minutes. PAC also displayed similar catalytic activity in a solution of the ecologically friendly ethanol, providing an alternative pathway to solid grinding. The added benefit of recyclability for up to four runs further solidified this green protocol with the challenge, however, of the lack of reactivity shown by ketones and aliphatic substrates [209].

**Scheme 77:**
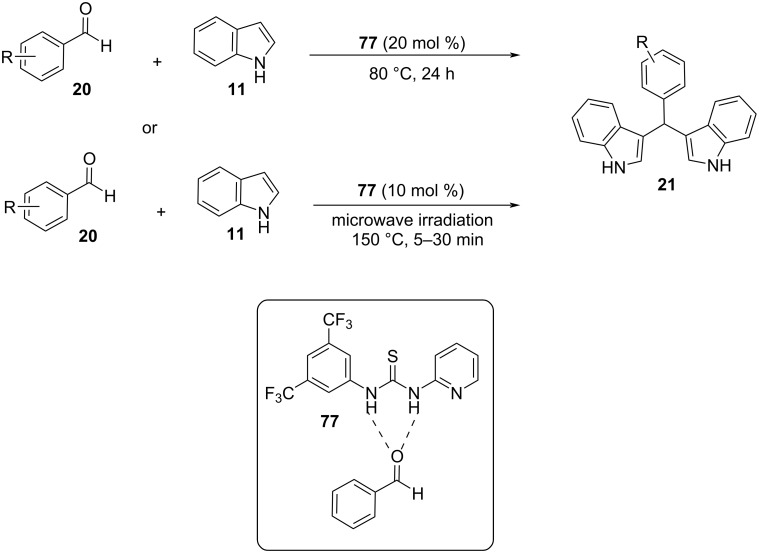
a) Reaction conditions employed by López and b) activation of the carbonyl group by thiourea.

In 2022, López and her co-workers developed an innovative organocatalytic methodology that employed thiourea, a hydrogen-donor catalyst for the activation of the carbonyl group (Scheme 77) [210]. Two different solvent-free catalytic pathways were tested with the first one utilizing conventional heating at 80 °C for 24 hours, while the second one included microwave irradiation (100 W) for 5 to 30 minutes with the oven set at 150 °C. While microwave irradiation accelerated reaction rates considerably, it slightly lowered product yields from 49–98% to 30–95%, due to local overheating being observed, which could lead to product decomposition. With conventional heating, the desired temperature was reached smoothly thus combatting this phenomenon. Nonetheless, a higher thiourea loading of 20 mol % was needed, compared to the 10 mol % when performing the reaction in the presence of microwave irradiation. Several substituted aromatic and heterocyclic aldehydes were screened successfully with the thiourea derivative **77** emerging as the most efficient organocatalyst. However, the need for heating in both pathways and the lack of ketones or aliphatic substrates utilized held back more widespread application of this protocol [210].

**Scheme 78:**
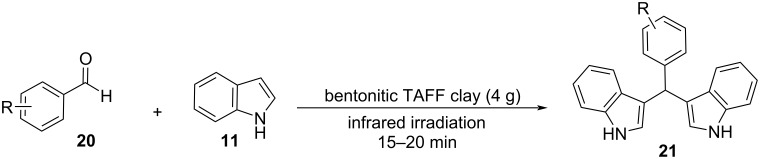
Infrared irradiation approach introduced by Luna-Mora and his research group.

In the same year, Luna-Mora and his research group published an unconventional approach for the synthesis of BIMs, making use of infrared technology, which is an underutilized tool in organic catalysis [211]. They specifically exploited the infrared irradiation (IR) zone of λ (1.5–3.0 μm) to attain the electrochemically excited species of the carbonyl group that is crucial for the formation of the C–C bond between aldehyde and indole. Bentonitic TAFF clay (4 g) was also employed for its intrinsic Brønsted–Lowry acid sites (BLAS), which promote it as a possible acid catalyst under IR irradiation. The implementation of the TAFF clay removes the need for both a solvent and catalyst, while offering low reaction times of 15–20 minutes (Scheme 78). Various substituted aromatic aldehydes and indoles managed to form the respective BIMs in satisfying yields, ranging from 60–96%, with electron-donating substituents elevating product conversion rates. Therefore, the mechanism of action of this protocol is based on the absorption of energy from the IR source, which fosters the formation of an excited complex that triggers a physisorption interaction with the BLAS leading to the initiation of the Friedel–Crafts alkylation [211].

#### Metal-organic frameworks

In 2017, metal-organic frameworks (MOFs) were introduced by de Campos and his co-workers as a unique alternative, for the heterogeneous catalysis of the Friedel–Crafts alkylation of aldehydes with indoles [212]. Specifically, a Co-MOF (based on CoCl_2_·6H_2_O) equipped with zwitterionic ligands, which coordinates to the metal center, causing polymerization of the structure, was implemented in the presence of sodium dodecyl sulfate (SDS), which acts as a surfactant, accelerating reaction rates. The optimum product yields were achieved, when water was utilized as the solvent at 50 °C with a catalyst loading of 5 mol % after a period of 2 hours. However, with the exception of benzaldehyde, all other substrates required a minimum of 24 hours to reach completion, yielding 78–97% of product, with no aliphatic substrates being mentioned in the substrate scope. Moreover, the use of conventional heating, the need for surfactants and the slow reaction rates presented issues that needed to be overcome [212].

**Scheme 79:**
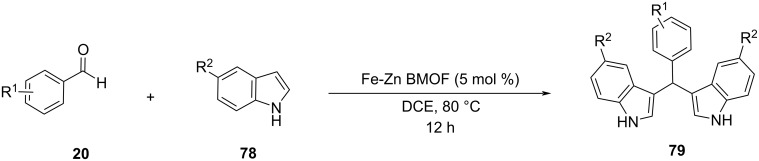
Synthesis of BIMs with the use of the Fe–Zn BMOF.

In 2019, Randhawa and her research team improved upon the use of MOFs for the synthesis of BIMs by implementing a Fe–Zn bimetallic MOF (BMOF), which displayed a considerably high surface area and was available through a simple solvothermal synthetic method (Scheme 79) [213]. When an amount of 10 mg of the Fe–Zn BMOF was employed, significant product yields were obtained (85–95%) with dichloroethane as the optimum solvent after a period of 12 hours. The reaction mixture was also heated at 80 °C to better facilitate the activation of the carbonyl group by the BMOF. Various aromatic and heteroaromatic substrates were screened with deactivated aldehydes (bearing electron-donating groups) displaying the lowest reaction rates. The BMOF also offered impressive recoverability options with no changes in catalytic activity being observed even after 7 reaction cycles. Nonetheless, the issue of the necessity of heating was not addressed [213].

One year later, a MOF consisting of Cu clusters (specifically Cu_3_(CO_2_)_6_(H_2_O)_2_) was introduced by Nguyen et al. as an alternative to the aforementioned MOFs [214]. What separated this methodology is the use of *m*-xylene as the reaction medium and the faster reaction rates, as only 2 hours were required to reach reaction completion for all different substrates. In place of conventional heating, sonication was employed to increase the MOF’s solubility with the product conversion rates being similar to Randhawa’s approach. Another differentiating factor is the lack of any notable fluctuations of reaction rates between the varied aromatic substrates, which enables the efficient synthesis of BIMs with pharmacologically relevant substitution patterns. The reusability of the metal-organic catalyst was also feasible for 6 cycles with no loss in catalytic performance, however, the lack of a green solvent and the necessity of sonication somewhat hindered the ecological aspects of this protocol [214].

## Conclusion

The Friedel–Crafts reaction between aldehydes and indoles represents a significant tool in the arsenal of organic chemists for the synthesis of BIMs. As more applications of BIMs in medicine and agriculture are discovered, new catalytic protocols are developed to facilitate their synthesis. However, while traditional approaches utilizing acidic catalysts are effective, the increased environmental awareness of our society renders the study of greener methodologies of pivotal importance. These studies focused on the employment of greener catalysts and solvents, in order to reduce toxic chemical waste and limit energy usage. Thus, in this review, emphasis was given on the greener and more ecological catalytic protocols developed over the years, while also highlighting the qualities that make BIMs such appealing pharmacological compounds.

## Data Availability

Data sharing is not applicable as no new data was generated or analyzed in this study.
